# A Systematic Review of the Outcomes of Utilization of Artificial Intelligence Within the Healthcare Systems of the Middle East: A Thematic Analysis of Findings

**DOI:** 10.1002/hsr2.70300

**Published:** 2024-12-24

**Authors:** Mohsen Khosravi, Seyyed Morteza Mojtabaeian, Emine Kübra Dindar Demiray, Burak Sayar

**Affiliations:** ^1^ Imam Hossein Hospital Shahroud University of Medical Sciences Shahroud Iran; ^2^ Department of Healthcare Services Management, School of Management and Medical Informatics Shiraz University of Medical Sciences Shiraz Iran; ^3^ Afyonkarahisar Public Hospital Infectious Diseases and Clinical Microbiology Afyonkarahisar Turkey; ^4^ Bitlis Eren University Vocational School of Social Sciences Bitlis Türkiye

**Keywords:** artificial intelligence, data mining, deep learning, health policy, machine learning

## Abstract

**Background and Aims:**

The rapid expansion of artificial intelligence (AI) within worldwide healthcare systems is occurring at a significant rate. In this context, the Middle East has demonstrated distinctive characteristics in the application of AI within the healthcare sector, particularly shaped by regional policies. This study examined the outcomes resulting from the utilization of AI within healthcare systems in the Middle East.

**Methods:**

A systematic review was conducted across several databases, including PubMed, Scopus, ProQuest, and the Cochrane Database of Systematic Reviews in 2024. The quality assessment of the included studies was conducted using the Authority, Accuracy, Coverage, Objectivity, Date, Significance checklist. Following this, a thematic analysis was carried out on the acquired data, adhering to the Boyatzis approach.

**Results:**

100 papers were included. The quality and bias risk of the included studies were delineated to be within an acceptable range. Multiple themes were derived from the thematic analysis including: “Prediction of diseases, their diagnosis, and outcomes,” “Prediction of organizational issues and attributes,” “Prediction of mental health issues and attributes,” “Prediction of polypharmacy and emotional analysis of texts,” “Prediction of climate change issues and attributes,” and “Prediction and identification of success and satisfaction among healthcare individuals.”

**Conclusion:**

The findings emphasized AI's significant potential in addressing prevalent healthcare challenges in the Middle East, such as cancer, diabetes, and climate change. AI has the potential to overhaul the healthcare systems. The findings also highlighted the need for policymakers and administrators to develop a concrete plan to effectively integrate AI into healthcare systems.

## Summary

- AI has a substantial potential in mitigating the challenges posed by cancer and diabetes in the Middle East.
- In situations where the healthcare system is strained, such as during a pandemic, AI can provide an effective solution.
- AI enhances the analysis of patient data, accelerating early diagnosis and treatment processes, while increasing the efficiency of healthcare delivery and optimal use of resources.
- AI can be utilized to confront the climate change and other similar challenges.
- There is a lack of studies concerning the effects of AI utilization in nonclinical facets of healthcare service delivery, encompassing areas such as human resource management and economics

## 1 Introduction

Artificial Intelligence (AI) denotes the advancement of computational systems capable of executing tasks traditionally reliant on human intelligence, such as pattern recognition, data‐driven learning, and decision‐making processes. Within the medical realm, AI stands poised to streamline workloads, elevate patient care standards, and optimize overall outcomes by aiding in critical functions such as treatment assessments and medical image analyses [1, 2].

Currently, the proliferation of AI within global healthcare systems is occurring at a notable pace. AI technologies are being progressively adopted across multiple medical domains, encompassing diagnostics, patient management, and hospital administration [3]. In this regard, the COVID‐19 pandemic has expedited the integration of digital technologies, including AI, within healthcare systems [4].

AI is widely regarded as a catalyst for enhancing various aspects of healthcare operations and service delivery. Forecasts indicate substantial cost savings of up to $150 billion annually in the United States by 2026. Additionally, AI facilitates a transition from a reactive healthcare paradigm to a proactive model, emphasizing health management as opposed to mere disease treatment [5].

AI possesses the capability to transform the landscape of healthcare by bolstering diagnostic precision, ameliorating patient prognoses, refining treatment strategies, mitigating healthcare expenditures, and tailoring medical interventions according to individual attributes such as genetic profiles, physiological metrics, and environmental determinants. AI's potential contributions span across a multitude of healthcare domains, encompassing administrative processes, medical imaging analyses, robotic surgical procedures, virtual assistant applications, clinical decision‐support systems, and precision medicine initiatives. This integration of AI‐enabled functionalities not only promises to create a healthcare framework characterized by enhanced efficiency and effectiveness in service delivery but also one that is highly adept at detecting false and unverified data [1, 6, 7, 8].

Multiple studies have indicated that although there exists a favorable disposition towards the imperative role of AI in the medical sector among the population residing in the Middle East, there remains a dearth of comprehension regarding its practical implementation and overarching significance [9, 10]. The Middle East constitutes a geographical region delineated to encompass the nations of Bahrain, Egypt, Iraq, Jordan, Kuwait, Lebanon, Oman, Qatar, Saudi Arabia, Syria, the United Arab Emirates, Yemen, Gaza, and the West Bank, as well as Iran and Turkey [11].

A study in the UAE found that physicians recognize both enabling and hindering factors in adopting AI applications for patient care, emphasizing the need for control, training, and interpretability of AI outcomes. Besides that, the Dubai Health Authority has implemented an AI policy to foster collaboration among healthcare stakeholders to enhance services [12]. In Saudi Arabia, the government is actively integrating AI into healthcare to improve service quality and address challenges like chronic diseases, although issues such as data quality and regulatory frameworks still need to be resolved [13]. In this regard, Saudi Arabia's Vision 2030 places significant emphasis on the integration of AI within the healthcare sector. This initiative is supported by the establishment of the Saudi Data and Artificial Intelligence Authority (SDAIA), which is tasked with leveraging data to enhance healthcare outcomes [14]. Therefore, Exploring and delineating the outcomes resulting from the integration of artificial intelligence across various facets of healthcare systems can serve as an effective strategy to bolster its utilization among the inhabitants of the region.

The Middle East faces several challenges in adopting AI in healthcare, including inadequate data quality and infrastructure, which hinder the implementation of AI solutions across medical specialties [3]. There is also a critical need for training healthcare professionals and raising awareness among providers and patients to foster acceptance of AI technologies [13, 15]. Additionally, concerns about equity highlight the risk that AI may exacerbate existing inequalities in healthcare access, necessitating careful consideration by policymakers to ensure that all populations benefit from these innovations [12, 16].

At the juncture of drafting this manuscript, no systematic reviews had been undertaken to scrutinize the ramifications stemming from the incorporation of AI within the healthcare systems of the Middle East. Nonetheless, within the existing literature, a solitary study emerged as a meta‐analysis, probing into the deployment of AI applications within the United Arab Emirates to mitigate and confront the COVID‐19 pandemic. The study revealed that AI applications proved efficacious in curbing the transmission of COVID‐19, surveilling adherence to imposed restrictions and preventive protocols, and furnishing remote healthcare services, thereby diminishing hospital admissions amidst periods of lockdown [17].

## 2 Methods

The study was qualitative in methodology conducted in 2024 adhering to the preferred reporting items for systematic reviews guidelines for systematic reviews [18].

### 2.1 Research Question

The research question was designed as: “What are the outcomes of the utilization of AI within healthcare systems in the Middle East?.”

### 2.2 Search Methodology

A comprehensive exploration was undertaken across various scholarly databases, including PubMed, Scopus, ProQuest, and the Cochrane database of systematic reviews, with the aim of discerning pertinent literature pertaining to the subject matter. We selected these databases due to their prominence and richness of data within the international stage. The search criteria were delineated into four distinct domains: Outcome, Artificial Intelligence, Health and Middle East. Initially, expansive terms were employed to heighten the search's sensitivity, while synonyms were integrated utilizing the “OR” operator. Subsequently, the “AND” operator was utilized to uphold precision and mitigate the inclusion of irrelevant studies. The outlined search methodology is detailed in Table 1.

**Table 1:** Search strategy.

Research question	What are the outcomes of utilization of Artificial Intelligence within the healthcare systems of the Middle East?
Key concepts or terms	Outcome, Artificial Intelligence, Health, Middle East
Databases or sources	Cochrane Library, PubMed, ProQuest, and Scopus.
Time‐period	2000‐2024
#1	outcome* OR Effect OR effects OR result* OR consequence* OR impact OR impacts OR influence OR influences
#2	artificial intelligence OR AI OR machine learning OR deep learning OR Data mining
#3	Health OR Healthcare
#4	Middle‐east* OR Bahrain* OR Cypr* OR Egypt* OR Iran* OR Leban* OR Syria* OR Iraq* OR Palestin* OR Jordan* OR Kuwait* OR Oman* OR Qatar* OR Turk* OR United Arab Emirates OR UAE OR Emirat* OR Yemen*
Final strategy	**#1 AND #2 AND #3 AND #4**

### 2.3 Inclusion/Exclusion Criteria

We included studies published in the English language between 2000 and 2024, focusing on the outcomes of AI utilization within healthcare systems in the Middle East. Studies were excluded if they failed to: (1) Address any data pertinent to the study's research question, (2) possess a title or abstract elucidating the research question of the study, or (3) contain a title, abstract, or full‐text that furnished any data concerning the research question of the study.

### 2.4 Screening and Data Gathering

Following a thorough examination of the studies, pertinent information was extracted using a structured data summarization and collection form. This template included essential details such as publication year, country of origin, study context, study type, AI type, and summary of the study. Summarization forms were diligently completed for each selected article. Subsequently, two researchers reviewed all completed forms and organized them into a table format. Finally, the third researcher contributed insights into any contradictory issues identified within the studies. The form was completed using Microsoft Word software, version 2020.

### 2.5 Quality Appraisal of Final Studies Using the AACODS Checklist

Two evaluators assessed the quality of all the incorporated studies using the Authority, Accuracy, Coverage, Objectivity, Date, Significance (AACODS) checklist. The AACODS checklist consisted of six questions [19]. During this process, a standardized scoring system was implemented, where a score of “Yes” corresponded to 2, “Can't Tell” to 1, and ‘No’ to 0. These scores ranged from 0 to 12, with higher scores denoting superior quality. The studies were then categorized into one of four groups based on their scores: very low quality (0–3), low quality (4–6), medium quality (7–9), and high quality (10–12). Only studies categorized as medium or high quality were deemed suitable for inclusion in the research endeavors. Any discrepancies between the two authors were resolved through discussion and consultation with a third party acting as an arbitrator. These procedures were repeated twice to ensure consistency.

### 2.6 Data Analysis

The data extracted from the preceding steps underwent analysis employing a qualitative thematic approach coupled with an inductive methodology. The Boyatzis method for thematic analysis was utilized in this analytical process [20]. The authors systematically reviewed all included studies and extractions to comprehensively comprehend the data, subsequently assigning initial codes to each significant extraction. During the process, a specific code was developed for the data, reflecting a unique outcome of artificial intelligence in healthcare. In the subsequent step, codes that exhibited a common concept were categorized together as sub‐themes. Furthermore, sub‐themes that shared a relatively similar context were consolidated under a single overarching theme. Before categorization into sub‐themes and main themes, all initial codes underwent thorough examination and finalization. Following this, sub‐themes and main themes were designated, elucidated, and organized in a tabular format. Furthermore, to ensure the validity and reliability of the analysis, the authors adhered to the Lincoln and Guba's criteria for qualitative research. These criteria encompassed four fundamental aspects: credibility, transferability, dependability, and confirmability of the qualitative content analysis process [21]. To ensure credibility, the authors conducted rigorous cross‐referencing of the codes with their respective original references on multiple occasions. To establish dependability, a pair of authors conducted the thematic analysis simultaneously and independently to identify any disparities in the results. Confirmability was safeguarded through cross‐examination of the themes, subthemes, and codes by the authors. Lastly, to enhance transferability, the authors ensured that the insights derived from the thematic analysis were expressed in a manner conducive to potential application across various contexts within the healthcare system.

## 3 Results

### 3.1 Systematic Review

**Figure 1:**
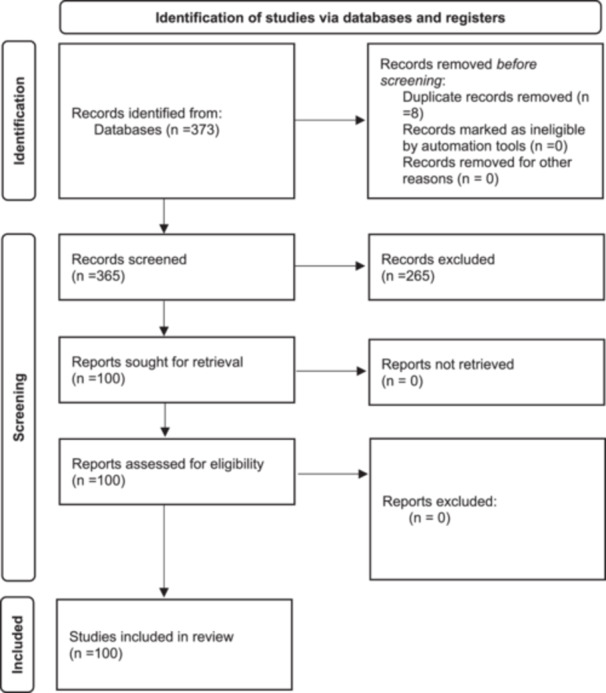
Preferred reporting items for systematic reviews diagram of the systematic review [18].

**Figure 2:**
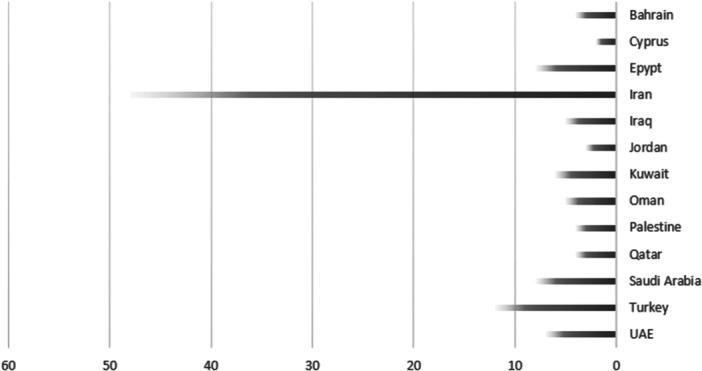
Countries in which the included studies were conducted.

The findings of the review revealed that among the 373 studies identified from the databases, 8 were found to be duplicates. Following the completion of the screening process, a total of 100 papers were ultimately chosen as the final selection for inclusion in the research (Figure 1). The delineation of the countries wherein the articles were conducted is presented in Figure 2. All of the studies included employed a quantitative approach and utilized machine learning techniques to analyze their data. Appendix 1 (Bibliography) presents further details regarding the characteristics of the included studies.

### 3.2 Quality Assessment

The quality assessment of the included studies revealed a high level of quality and a low risk of bias. The results of the assessment indicated that the question concerning the dates of the studies received the lowest score, suggesting that majority of the studies might be relatively old. Additional information regarding the results of the quality assessment for the included studies can be found in Appendix 2 (Quality Assessment).

### 3.3 Thematic Analysis

**Figure 3:**
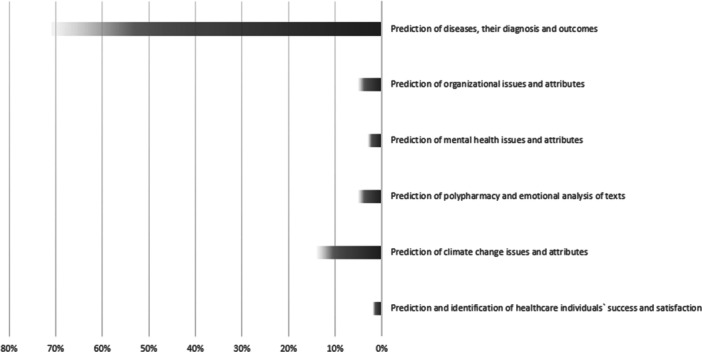
Distribution of each theme of the thematic analysis among the included studies.

As delineated in Table 2, the outcomes of the thematic analysis conducted on the data obtained from the included studies were classified into six primary themes. The themes encompassed items including “Prediction of diseases, their diagnosis and outcomes,” “Prediction of organizational issues and attributes,” “Prediction of mental health issues and attributes,” “Prediction of polypharmacy and emotional analysis of the texts,” “Prediction of climate changes issues and attributes,” and “Prediction and identification of healthcare individuals success and satisfaction.” The distribution of each theme among the included studies is illustrated in Figure 3.

**Table 2:** Thematic analysis of the data acquired from the included studies.

Theme	Subtheme	Context	Reference
Prediction of diseases, their diagnosis and outcomes	Prediction of diabetes, diabetic nephropathy, ulcers and insulin resistance	Iran, Kuwait, Oman, Egypt	Abdesselam et al. [22, 23, 24, 25, 26, 27, 28, 29]
	Detection of individuals at risk of cancer, its diagnosis, recurrence, metastasis and patient survival	Iran, Turkey, Egypt	Afrash et al. [30, 31, 32, 33, 34, 35, 36, 37, 38, 39, 40, 41, 42, 43, 44]
	Prediction of necessity of interventions for brain disorders	Iran	Habibzadeh et al. [45, 46]
	Predicting of fetal, postpartum disorders, newborn birth weight, neonatal mortality and implantation outcomes for individual embryos	Turkey, Oman UAE, Iran	Akbulut et al. [47, 48, 49, 50, 51, 52]
	Prediction of β‐thalassemia	Palestine	AlAgha et al. [53]
	Prediction of multimorbidity	Saudi Arabia, Kuwait	Albagmi et al. [25, 54]
	Prediction of the number of covid‐19 patients, its severity, outcomes and risk mapping	Saudi Arabia, UAE, Egypt, Iran, Bahrain, Kuwait, Qatar, Oman, Iraq,	Alrajhi et al. [55, 56, 57, 58, 59, 60, 61, 62, 63, 64, 65, 66, 67, 68, 69, 70, 71]
	Prediction and diagnosis of tuberculosis, brucellosis and other microbiological diseases	Iran, Jordan	Amoori et al. [72, 73, 74, 75, 76, 77]
	Prediction of cardiac and coronary diseases	Bahrain, Kuwait, Oman, UAE, Turkey, Qatar	AlKaabi et al. [25, 78, 79, 80]
	Prediction of systemic lupus erythematosus	Oman	AlShareedah et al. [81]
	Prediction of outcome in trauma patients	Iran	Hassanzadeh et al. [82, 83]
	Prediction of respiratory syndromes spatial‐temporal patterns and their recovery	Saudi Arabia, Iran	John, Shaiba [84, 85]
	Detecting vitamin D status	Cyprus	Sancar, Tabrizi [86]
	Detection of rare thyroid nodules	Turkey	Turk et al. [87]
	Detection of dental and oral disorders	Iran, Cyprus, Turkey	ForouzeshFar et al. [88, 89, 90]
	Prediction of patient‐activation level	Turkey	Demiray et al. [91]
	Automated quality assessment of telehealth services	Middle East	Habib et al. [92]
Prediction of organizational issues and attributes	Optimal Co‐insurance Estimation	Iran	Momahhed et al. [93]
	Forecasting patient demand	Iran	Soltani et al. [94]
	Forecasting Medical Equipment Demand	Turkey	Koç, Türkoğlu [95]
	Optimizing care flows and processes	Egypt	Rashed et al. [96]
	Prediction of work stress	Egypt	Torad et al. [97]
Prediction of mental health issues and attributes	Prediction of Suicide Ideation/Behavior	Middle East	Naghavi et al. [98]
	Prediction of Maternal Depression and Anxiety	Jordan, Palestine, Lebanon, Saudi Arabia, Bahrain	Qasrawi et al. [99]
	Detection of children's mental health and cognitive development	Palestine	Qasrawi et al. [100]
Prediction of polypharmacy and emotional analysis of texts	Prediction of polypharmacy	Iran	Seyedtabib, Kamyari [101]
	Emotional analysis of texts	Saudi Arabia, Iran, Turkey	Alshalan et al. [102, 103, 104, 105]
Prediction of climate change issues and attributes	Prediction of Food Insecurity	Jordan, Palestine, Lebanon, Saudi Arabia, Bahrain	Qasrawi et al. [106]
	Prediction the concentration of nitrogen dioxide (NO2)	UAE, Iran	Al Yammahi, Aung [107, 108, 109]
	Prediction of particulate air pollution	Iran, Iraq, Kuwait	Gholami et al. [110, 111, 112, 113, 114]
	Prediction of heavy metal pollution	Turkey	Günal et al. [115]
	Prediction of salt concentration in water	Iran	Jamei et al. [116]
	Prediction of water pollution level	Iran	Mohammadpour et al. [117]
	Prediction of dust susceptibility	Iran	Razavi‐Termeh et al. [118]
	Estimating soil organic carbon (SOC)	Iran	Zhang et al. [119]
Prediction and identification of healthcare individuals' success and satisfaction	Prediction of healthcare student success	Qatar	Hammoudi Halat et al. [120]
	Identification of key determinants of patient satisfaction	UAE	Simsekler et al. [121]

#### 3.3.1 Prediction of Diseases, Their Diagnosis and Outcomes

Approximately 71% of the included studies addressed this overarching theme, which encompasses various sub‐themes. The sub‐themes included the prediction of diabetes, diabetic nephropathy, ulcers, and insulin resistance; the detection of individuals at risk of cancer, its diagnosis, recurrence, metastasis, and patient survival; the prediction of necessity for interventions concerning brain disorders; forecasting fetal and postpartum disorders, newborn birth weight, neonatal mortality, and implantation outcomes for individual embryos; the prediction of β‐thalassemia; prognostication of multimorbidity; forecasting the number of COVID‐19 patients, its severity, outcomes, and risk mapping; the prediction and diagnosis of tuberculosis, brucellosis, and other microbiological diseases; anticipation of cardiac and coronary diseases; prediction of systemic lupus erythematosus; prognostication of outcomes in trauma patients; prediction of respiratory syndrome spatial‐temporal patterns and their recovery; detection of vitamin D status; identification of rare thyroid nodules; detection of dental and oral disorders; prediction of patient‐activation levels; and automated quality assessment of telehealth services.

#### 3.3.2 Prediction of Organizational Issues and Attributes

Addressed by approximately 5% of the studies, this theme comprised of several sub‐themes including: optimal co‐insurance estimation, forecasting patient demand, forecasting medical equipment demand, optimizing care flows and processes, and prediction of work stress.

#### 3.3.3 Prediction of Mental Health Issues and Attributes

Addressed by approximately 3% of the studies, this theme included multiple subthemes. The sub‐themes included the prediction of suicide ideation/behavior, maternal depression and anxiety, as well as the detection of children's mental health and cognitive development.

#### 3.3.4 Prediction of Polypharmacy and Emotional Analysis of the Texts

This theme was addressed by approximately 5% of the studies. The corresponding sub‐themes for this theme included prediction of polypharmacy, and emotional analysis of the texts.

#### 3.3.5 Prediction of Climate Changes Issues and Attributes

Addressed by approximately 14% of the studies, this theme included several subthemes. The sub‐themes included the prediction of food insecurity, concentration of nitrogen dioxide, particulate air pollution, heavy metal pollution, salt concentration in water, water pollution level, dust susceptibility, and estimating soil organic carbon.

#### 3.3.6 Prediction and Identification of Healthcare Individuals' Success and Satisfaction

This theme addressed by roughly 2% of the studies. The theme comprised of multiple sub‐themes including prediction of healthcare student success, and Identification of key determinants of patient satisfaction.

## 4 Discussion

As delineated by the findings of the study outlined in the preceding section, the studies conducted in Iran constituted 48% of the total studies, surpassing those conducted in other countries. Meanwhile, Turkey ranked second, with a mere 12% share of the included studies.

The substantial utilization of AI within Iranian literature found within the current study presented an intriguing departure from prior research indicating a moderate rather than high level of familiarity with AI among Iranian healthcare professionals, alongside a moderately favorable disposition towards its application in medicine. Nonetheless, there exists a cautious attitude among Iranian healthcare staff regarding the expanding role of AI in the medical domain. Moreover, it is highlighted that Iranian healthcare professionals identify the enhancement of diagnostic test accuracy, drug interaction identification, and medical test and imaging analysis as the principal areas where AI can be effectively employed in medicine [122]. Another study indicated that Iranian experts regard clinical decision‐making, medical diagnosis, medical procedures, and patient‐centered care as the primary benefits of ChatGPT, a prominent AI model in the healthcare sector. It was further determined that ChatGPT demonstrates the highest level of usefulness in the domains of information and infrastructure as well as information and communication technologies [123].

As delineated in the findings of the study, the outcomes of utilization of AI in healthcare systems of the middle east could be classified into 6 major themes. The results outlined that the thematic domain of ‘prediction of diseases, their diagnosis, and outcomes’ held the predominant share among the themes in terms of citation within the included studies, amounting to 71% of the included studies. Following this, “prediction of climate change issues and attributes” secured the second position with a 14% share, while “prediction and identification of healthcare individuals” success and satisfaction' garnered the lowest citation among the included studies, representing only 2% of the studies.

Multiple studies with the objective of prioritizing areas for the utilization of artificial AI within healthcare services have outlined its potential in enhancing disease diagnosis and treatment recommendations. In this regard, AI's capability to analyze extensive datasets enables the identification of patterns and surpasses human performance in disease diagnosis, treatment selection, and clinical laboratory testing [124, 125]. This implication aligns with the findings of our study, which identified the domain of predicting diseases, their diagnosis, and outcomes as having the highest citation rate among the included studies.

Within the thematic domain of “prediction of diseases, their diagnosis, and outcomes,” the subtheme focused on “prediction of the number of COVID‐19 patients, its severity, outcomes, and risk mapping” emerged as the most significant contributor among the included studies, constituting approximately 17% of the studies. Conversely, the subtheme concerning “detection of individuals at risk of cancer, its diagnosis, recurrence, metastasis, and patient survival” occupied the second position in terms of contribution, comprising 15% of the included studies.

Multiple studies have underscored the noteworthy impact of the COVID‐19 pandemic on the increased utilization of AI within healthcare systems [126, 127, 128]. This phenomenon elucidates the rationale behind the substantial number of studies documented in our research, which reported on the utilization of AI during the COVID‐19 pandemic. In this context, artificial AI has been deployed during the pandemic to mitigate human‐to‐human contact and minimize exposure risk through the assistance of robotics [127]. Additionally, AI has played a role in screening, diagnosing, prognosticating, and treating COVID‐19 cases [128, 129]. Furthermore, AI has the potential to aid in disease surveillance, contact tracing, drug discovery, and providing clinical decision support [130].

The prevailing condition of cancer within the Middle Eastern realm, specifically in the Arab nations, is a matter of substantial apprehension. Recent research indicates that cancer constitutes a prominent public health challenge in the Middle East, where colorectal cancer ranks as the second most prevalent form of cancer and the third leading cause of mortality in the region [131]. This phenomenon elucidates the rationale behind the substantial adoption AI within the healthcare services catering to cancer patients reported by a considerable portion (17%) of the studies included within this paper [55, 56, 57, 58, 59, 60, 61, 62, 63, 64, 65, 66, 67, 68, 69, 70, 71]. It underscores the considerable potential of AI in addressing the challenge of cancer in the Middle East, contingent upon the formulation and execution of a meticulous strategic plan.

The Middle East exhibits the highest global prevalence of diabetes, accompanied by the second‐highest rate of increase worldwide. This surge is fueled by factors such as obesity, sedentary lifestyles, urbanization, and inadequate dietary practices [132, 133, 134, 135]. In this context, it is noteworthy that a substantial proportion (8%) of the studies reviewed in this paper documented the effective application of AI in predicting diabetes, diabetic nephropathy, ulcers, and insulin resistance among patients [22, 23, 24, 25, 26, 27, 28, 29]. This discovery also holds significant implications, indicating the potential of AI utilization in enhancing the condition of diabetic patients in Middle Eastern countries.

Another finding reported by our study pertained to the notable outcomes associated with the application of AI in predicting climate change issues and attributes, as reported by 14% of the studies included in this review [100, 107, 108, 109, 110, 111, 112, 113, 114, 115, 116, 117, 118, 119]. The Middle East region is recognized as one of the most susceptible to the repercussions of climate change. It is undergoing a pronounced warming trend characterized by prolonged and hotter summers, heightened occurrence and severity of heatwaves, and a reduction in precipitation and water resources [136, 137]. Consequently, the depiction of successful and noteworthy utilization of AI concerning climate change within this paper constitutes another substantial implication.

## 5 Limitations and Implications

The study's limitation resided in its exclusive inclusion of English‐language papers, thereby overlooking those composed in the native languages of each respective country. This oversight carries significant implications for healthcare administrators, policymakers, and prospective researchers within the field. This research emphasized the substantial potential of AI in mitigating the challenges posed by cancer and diabetes in the Middle East, subject to the development and implementation of a comprehensive strategic framework. In situations where the healthcare system is strained, such as during a pandemic, AI can provide an effective solution. AI can analyze patient data quickly and accurately, accelerating early diagnosis and treatment processes. Additionally, it enhances the efficiency of healthcare delivery and supports the optimal use of resources. Another implication of the study was the depiction of successful and noteworthy utilization of AI concerning climate change which can be utilized by the beneficiaries. Additionally, a dearth of research exists concerning the effects of AI utilization in nonclinical facets of healthcare service delivery, encompassing areas such as human resource management and economics, which can be an implication for future researchers. Lastly, Due to the limited scope of this study's manuscript, we were unable to address several important issues concerning the challenges and barriers to the utilization of AI in the Middle East. These issues could be suggested as implications for future researchers, who could explore challenges of AI utilization such as ethics, infrastructure, and technical barriers. Addressing these topics could yield valuable insights for stakeholders in the region.

## 6 Conclusion

This study identified several significant outcomes associated with the utilization of AI within healthcare systems in the Middle East. Among the studies reviewed, Iran demonstrated the highest level of contribution, followed by Turkey. The most frequently reported domains included disease prediction, diagnosis, and subsequent outcomes, while the prediction of climate change issues also emerged as a notable outcome of AI application in healthcare. The findings emphasized the considerable potential of AI in addressing prevalent healthcare challenges in the region, including cancer, diabetes, and climate change. These findings highlighted the significant opportunity for the utilization of AI in healthcare, contingent upon the development of a concrete plan by policymakers and administrators within the region's healthcare systems.

## Author Contributions

M.K. conducted the search within the databases, wrote the introduction, results, methods and discussion sections. M.K. and S.M.M. extracted the data and conducted the analysis; S.M.M. consulted with M.K. during each phase of the study. E.K.D.D. and B.S. revised the manuscript.

## Ethics Statement

The authors have nothing to report.

## Conflicts of Interest

The authors declare no conflicts of interest.

### 1 Transparency Statement

MK affirms that this manuscript is an honest, accurate, and transparent account of the study being reported; that no important aspects of the study have been omitted; and that any discrepancies from the study as planned (and, if relevant, registered) have been explained.

## Supporting information

Supporting information.

Supporting information.

## Data Availability

M.K. had full access to all of the data in this study and takes complete responsibility for the integrity of the data and the accuracy of the data analysis. The study data is available through contacting M.K. (Corresponding author).

## References

[1] A. Amisha , P. Malik , M. Pathania , and V. Rathaur , “Overview of Artificial Intelligence in Medicine,” Journal of Family Medicine and Primary Care 8, no. 7 (2019): 2328–2331, 10.4103/jfmpc.jfmpc_440_19. PMC6691444 31463251

[2] L. Kiester and C. Turp , “Artificial Intelligence Behind the Scenes: PubMed's Best Match Algorithm,” Journal of the Medical Library Association 110, no. 1 (2022): 15–22, 10.5195/jmla.2022.1236. 35210958 PMC8830327

[3] V. Zuhair , A. Babar , R. Ali , et al., “Exploring the Impact of Artificial Intelligence on Global Health and Enhancing Healthcare in Developing Nations,” Journal of Primary Care & Community Health 15 (2024): 21501319241245847, 10.1177/21501319241245847. PMC11010755 38605668

[4] T. D. Hadley , R. W. Pettit , T. Malik , A. A. Khoei , and H. M. Salihu , “Artificial Intelligence in Global Health ‐A Framework and Strategy for Adoption and Sustainability,” International Journal of Maternal and Child Health and AIDS (IJMA) 9, no. 1 (2020): 121–127, 10.21106/ijma.296. 32123635 PMC7031870

[5] A. Bohr and K. Memarzadeh , “The Rise of Artificial Intelligence in Healthcare Applications,” Artificial Intelligence in Healthcare. Published ahead of print, June 26, 2020, 10.1016/b978-0-12-818438-7.00002-2.

[6] Z. Amiri , A. Heidari , N. J. Navimipour , M. Esmaeilpour , and Y. Yazdani , “The Deep Learning Applications in Iot‐Based Bio‐ and Medical Informatics: A Systematic Literature Review,” Neural Computing and Applications 36, no. 11 (2024): 5757–5797, 10.1007/s00521-023-09366-3.

[7] A. S. Ahuja , “The Impact of Artificial Intelligence in Medicine on the Future Role of the Physician,” PeerJ 7 (2019): e7702, 10.7717/peerj.7702. 31592346 PMC6779111

[8] A. Heidari , N. Jafari Navimipour , H. Dag , and M. Unal , “Deepfake Detection Using Deep Learning Methods: A Systematic and Comprehensive Review,” WIREs Data Mining and Knowledge Discovery 14, no. 2 (2024): e1520, 10.1002/widm.1520.

[9] W. Al‐Qerem , J. Eberhardt , A. Jarab , et al., “Exploring Knowledge, Attitudes, and Practices Towards Artificial Intelligence Among Health Professions’ Students in Jordan,” BMC Medical Informatics and Decision Making 23, no. 1 (2023): 288, 10.1186/s12911-023-02403-0. 38098095 PMC10722664

[10] S. Swed , H. Alibrahim , N. K. H. Elkalagi , et al., “Knowledge, Attitude, and Practice of Artificial Intelligence Among Doctors and Medical Students in Syria: A Cross‐Sectional Online Survey,” Frontiers in Artificial Intelligence 5 (2022): 1011524, 10.3389/frai.2022.1011524. 36248622 PMC9558737

[11] A. R. Omran and F. Roudi , “The Middle East Population Puzzle,” Population Bulletin 48, no. 1 (1993): 1–40. 12318382

[12] T. Mansour and M. Bick , “How Can Physicians Adopt AI‐Based Applications in the United Arab Emirates to Improve Patient Outcomes,” Digital Health 10 (2024): 20552076241284936, 10.1177/20552076241284936. 39351313 PMC11440542

[13] A. Saeed , A. Bin Saeed , and F. A. AlAhmri , “Saudi Arabia Health Systems: Challenging and Future Transformations With Artificial Intelligence,” Cureus 15, no. 4 (2023): e37826, 10.7759/cureus.37826. 37214025 PMC10197987

[14] Z. A. Memish , M. M. Altuwaijri , A. H. Almoeen , and S. M. Enani , “The Saudi Data & Artificial Intelligence Authority (SDAIA) Vision: Leading the Kingdom's Journey Toward Global Leadership,” Journal of Epidemiology and Global Health 11, no. 2 (2021): 140–142, 10.2991/jegh.k.210405.001. 33876596 PMC8242113

[15] H. A. Amin and T. M. Alanzi , “Utilization of Artificial Intelligence (AI) in Healthcare Decision‐Making Processes: Perceptions of Caregivers in Saudi Arabia,” Cureus 16, no. 8 (2024): e67584, 10.7759/cureus.67584. 39310597 PMC11416819

[16] A. El‐Sayed , S. Salman , and L. Alrubaiy , “The Adoption of Artificial Intelligence Assisted Endoscopy in the Middle East: Challenges and Future Potential,” Translational Gastroenterology and Hepatology 8 (2023): 42, 10.21037/tgh-23-37. 38021356 PMC10643188

[17] H. Haneya , D. AlKaf , F. Bajammal , and T. Brahimi , “A Meta‐Analysis of Artificial Intelligence Applications for Tracking COVID‐19: The Case of the U.A.E,” Procedia Computer Science 194 (2021): 180–189, 10.1016/j.procs.2021.10.072. 34876933 PMC8641301

[18] M. J. Page , J. E. McKenzie , P. M. Bossuyt , et al., “The PRISMA 2020 Statement: An Updated Guideline for Reporting Systematic Reviews,” BMJ 372 (2021): n71, 10.1136/bmj.n71. 33782057 PMC8005924

[19] J. Tyndall , AACODS (Authority, Accuracy, Coverage, Objectivity, Date, Significance) Checklist (Flinders, Australia: Flinders University, 2010).

[20] R. Boyatzis , Transforming Qualitative Information: Thematic Analysis and Code Development (Sage, 1998).

[21] R. Forero , S. Nahidi , J. De Costa , et al., “Application of Four‐Dimension Criteria to Assess Rigour of Qualitative Research in Emergency Medicine,” BMC Health Services Research 18, no. 1 (2018): 120, 10.1186/s12913-018-2915-2. 29454350 PMC5816375

[22] K. Al Sadi and W. Balachandran , “Revolutionizing Early Disease Detection: A High‐Accuracy 4D CNN Model for Type 2 Diabetes Screening in Oman,” Bioengineering 10, no. 12 (December 2023): 1420, 10.3390/bioengineering10121420. 38136011 PMC10740649

[23] H. Esmaeily , M. Tayefi , M. Ghayour‐Mobarhan , and A. Amirabadizadeh , “Comparing Three Data Mining Algorithms for Identifying the Associated Risk Factors of Type 2 Diabetes,” Iranian Biomedical Journal 22, no. 5 (2018): 303–311, 10.29252/ibj.22.5.303. 29374085 PMC6058191

[24] B. Farran , R. AlWotayan , H. Alkandari , D. Al‐Abdulrazzaq , A. Channanath , and T. A. Thanaraj , “Use of Non‐Invasive Parameters and Machine‐Learning Algorithms for Predicting Future Risk of Type 2 Diabetes: A Retrospective Cohort Study of Health Data From Kuwait,” Frontiers in Endocrinology 10 (2019): 624, 10.3389/fendo.2019.00624. 31572303 PMC6749017

[25] B. Farran , A. M. Channanath , K. Behbehani , and T. A. Thanaraj , “Predictive Models to Assess Risk of Type 2 Diabetes, Hypertension and Comorbidity: Machine‐Learning Algorithms and Validation Using National Health Data From Kuwait–A Cohort Study,” BMJ Open 3, no. 5 (2013): e002457, 10.1136/bmjopen-2012-002457. PMC3657675 23676796

[26] S. M. Hosseini Sarkhosh , A. Esteghamati , M. Hemmatabadi , and M. Daraei , “Predicting Diabetic Nephropathy in Type 2 Diabetic Patients Using Machine Learning Algorithms,” Journal of Diabetes & Metabolic Disorders 21, no. 2 (2022): 1433–1441, 10.1007/s40200-022-01076-2. 36404838 PMC9672147

[27] A. Abdesselam , H. Zidoum , F. Zadjali , et al., “Estimate of the HOMA‐IR Cut‐Off Value Identifying Subjects at Risk of Insulin Resistance Using a Machine Learning Approach,” Sultan Qaboos University Medical Journal [SQUMJ] 21, no. 4 (2021): 604–612, 10.18295/squmj.4.2021.030. 34888081 PMC8631209

[28] K. M. Mousa , F. A. Mousa , H. S. Mohamed , and M. M. Elsawy , “Prediction of Foot Ulcers Using Artificial Intelligence for Diabetic Patients at Cairo University Hospital, Egypt,” SAGE open nursing 9 (2023): 23779608231185873, 10.1177/23779608231185873. 37435577 PMC10331222

[29] H. Shojaee‐Mend , F. Velayati , B. Tayefi , and E. Babaee , “Prediction of Diabetes Using Data Mining and Machine Learning Algorithms: A Cross‐Sectional Study,” Healthcare Informatics Research 30, no. 1 (2024): 73–82, 10.4258/hir.2024.30.1.73. 38359851 PMC10879823

[30] M. R. Afrash , A. Bayani , M. Shanbehzadeh , M. Bahadori , and H. Kazemi‐Arpanahi , “Developing the Breast Cancer Risk Prediction System Using Hybrid Machine Learning Algorithms,” Journal of Education and Health Promotion 11 (2022): 272, 10.4103/jehp.jehp_42_22. 36325225 PMC9621357

[31] A. Ansaripour , K. Zendehdel , N. Tadayon , F. Sadeghi , C. A. Uyl‐de Groot , and W. K. Redekop , “Use of Data‐Mining to Support Real‐World Cost Analyses: An Example Using HER2‐positive Breast Cancer in Iran,” PLoS One 13, no. 10 (2018): e0205079, 10.1371/journal.pone.0205079. 30273393 PMC6166984

[32] A. Choudhury , “Predicting Cancer Using Supervised Machine Learning: Mesothelioma,” Technology and Health Care 29, no. 1 (2021): 45–58, 10.3233/thc-202237. 32568137

[33] S. Dehdar , K. Salimifard , R. Mohammadi , et al., “Applications of Different Machine Learning Approaches in Prediction of Breast Cancer Diagnosis Delay,” Frontiers in Oncology 13 (2023): 1103369, 10.3389/fonc.2023.1103369. 36874113 PMC9978377

[34] M. Dianati‐Nasab , K. Salimifard , R. Mohammadi , et al., “Machine Learning Algorithms to Uncover Risk Factors of Breast Cancer: Insights From a Large Case‐Control Study,” Frontiers in Oncology 13 (2023): 1276232, 10.3389/fonc.2023.1276232. 38425674 PMC10903343

[35] M. Emiroglu , H. Esin , M. Erdogan , et al., “National Study on Use of Artificial Intelligence in Breast Disease and Cancer,” Bratislavske Lekarske Listy 123, no. 3 (2022): 191–196, 10.4149/bll_2022_032. 35343751

[36] A. F , S. C., and A. L., “Supervised Algorithms of Machine Learning for the Prediction of Cervical Cancer,” Journal of Biomedical Physics & Engineering 10, no. 4 (2020): 513–522, 10.31661/jbpe.v0i0.1912-1027. 32802799 PMC7416093

[37] L. H. A. Fryan and M. B. Alazzam , “Survival Analysis of Oncological Patients Using Machine Learning Method,” Healthcare 11, no. 1 (December 2022): 80, 10.3390/healthcare11010080. 36611540 PMC9818920

[38] G. Mohammadi , M. Azizmohammad Looha , M. A. Pourhoseingholi , et al., “Classification and Diagnostic Prediction of Colorectal Cancer Mortality Based on Machine Learning Algorithms: A Multicenter National Study,” Asian Pacific Journal of Cancer Prevention 25, no. 1 (2024): 333–342, 10.31557/apjcp.2024.25.1.333. 38285801 PMC10911721

[39] K. Mohammadnezhad , M. R. Sahebi , S. Alatab , and A. Sadjadi , “Modeling Epidemiology Data With Machine Learning Technique to Detect Risk Factors for Gastric Cancer,” Journal of gastrointestinal cancer 55 (2024): 287–296, 10.1007/s12029-023-00952-1. 37428282

[40] A. Mosayebi , B. Mojaradi , A. Bonyadi Naeini , and S. H. Khodadad Hosseini , “Modeling and Comparing Data Mining Algorithms for Prediction of Recurrence of Breast Cancer,” PLoS One 15, no. 10 (2020): e0237658, 10.1371/journal.pone.0237658. 33057328 PMC7561198

[41] R. Nopour , “Prediction of Five‐Year Survival Among Esophageal Cancer Patients Using Machine Learning,” Heliyon 9, no. 12 (2023): e22654, 10.1016/j.heliyon.2023.e22654. 38125437 PMC10730993

[42] R. Safdari , E. Maserat , H. Asadzadeh Aghdaei , A. H. Javan Amoli , and H. Mohaghegh Shalmani , “Person Centered Prediction of Survival in Population Based Screening Program by an Intelligent Clinical Decision Support System,” Gastroenterology and Hepatology From Bed to Bench 10, no. 1 (2017): 60–65. 28331566 PMC5346826

[43] G. I. Sayed , M. Solyman , G. El Gedawy , Y. S. Moemen , H. Aboul‐Ella , and A. E. Hassanien , “Circulating miRNA's Biomarkers for Early Detection of Hepatocellular Carcinoma in Egyptian Patients Based on Machine Learning Algorithms,” Scientific Reports 14, no. 1 (2024): 4989, 10.1038/s41598-024-54795-2. 38424116 PMC10904762

[44] R. Talebi , C. A. Celis‐Morales , A. Akbari , A. Talebi , N. Borumandnia , and M. A. Pourhoseingholi , “Machine Learning‐Based Classifiers to Predict Metastasis in Colorectal Cancer Patients,” Frontiers in Artificial Intelligence 7 (2024): 1285037, 10.3389/frai.2024.1285037. 38327669 PMC10847339

[45] A. Habibzadeh , S. Khademolhosseini , A. Kouhpayeh , et al., “Machine Learning‐Based Models to Predict the Need for Neurosurgical Intervention After Moderate Traumatic Brain Injury,” Health Science Reports 6, no. 11 (2023): e1666, 10.1002/hsr2.1666. 37908638 PMC10613807

[46] A. A. Kashef , T. Khatibi , and A. Mehrvar , “Prediction of Cranial Radiotherapy Treatment in Pediatric Acute Lymphoblastic Leukemia Patients Using Machine Learning: A Case Study at MAHAK Hospital,” Asian Pacific Journal of Cancer Prevention 21, no. 11 (2020): 3211–3219, 10.31557/apjcp.2020.21.11.3211. 33247677 PMC8033115

[47] A. Akbulut , E. Ertugrul , and V. Topcu , “Fetal Health Status Prediction Based on Maternal Clinical History Using Machine Learning Techniques,” Computer Methods and Programs in Biomedicine 163 (2018): 87–100, 10.1016/j.cmpb.2018.06.010. 30119860

[48] W. Khan , N. Zaki , M. M. Masud , et al., “Infant Birth Weight Estimation and Low Birth Weight Classification in United Arab Emirates Using Machine Learning Algorithms,” Scientific Reports 12, no. 1 (2022): 12110, 10.1038/s41598-022-14393-6. 35840605 PMC9287292

[49] V. Mehrnoush , A. Ranjbar , M. V. Farashah , F. Darsareh , M. Shekari , and M. S. Jahromi , “Prediction of Postpartum Hemorrhage Using Traditional Statistical Analysis and a Machine Learning Approach,” AJOG Global Reports 3, no. 2 (2023): 100185, 10.1016/j.xagr.2023.100185. 36935935 PMC10020099

[50] A. Ranjbar , F. Montazeri , M. V. Farashah , V. Mehrnoush , F. Darsareh , and N. Roozbeh , “Machine Learning‐Based Approach for Predicting Low Birth Weight,” BMC Pregnancy and Childbirth 23, no. 1 (2023): 803, 10.1186/s12884-023-06128-w. 37985975 PMC10662167

[51] A. Sheikhtaheri , M. R. Zarkesh , R. Moradi , and F. Kermani , “Prediction of Neonatal Deaths in Nicus: Development and Validation of Machine Learning Models,” BMC Medical Informatics and Decision Making 21, no. 1 (2021): 131, 10.1186/s12911-021-01497-8. 33874944 PMC8056638

[52] A. Uyar , A. Bener , and H. N. Ciray , “Predictive Modeling of Implantation Outcome in an In Vitro Fertilization Setting: An Application of Machine Learning Methods,” Medical Decision Making 35, no. 6 (2015): 714–725, 10.1177/0272989x14535984. 24842951

[53] A. S. AlAgha , H. Faris , B. H. Hammo , and A. M. Al‐Zoubi , “Identifying β‐thalassemia Carriers Using a Data Mining Approach: The Case of the Gaza Strip, Palestine,” Artificial Intelligence in Medicine 88 (2018): 70–83, 10.1016/j.artmed.2018.04.009. 29730048

[54] F. M. Albagmi , M. Hussain , K. Kamal , et al., “Predicting Multimorbidity Using Saudi Health Indicators (Sharik) Nationwide Data: Statistical and Machine Learning Approach,” Healthcare 11, no. 15 (2023): 2176, 10.3390/healthcare11152176. 37570417 PMC10418949

[55] A. A. Alrajhi , O. A. Alswailem , G. Wali , et al., “Data‐Driven Prediction for COVID‐19 Severity in Hospitalized Patients,” International Journal of Environmental Research and Public Health 19, no. 5 (2022): 2958, 10.3390/ijerph19052958. 35270653 PMC8910504

[56] A. AlShehhi , T. M. Almansoori , A. R. Alsuwaidi , and H. Alblooshi , “Utilizing Machine Learning for Survival Analysis to Identify Risk Factors for COVID‐19 Intensive Care Unit Admission: A Retrospective Cohort Study From the United Arab Emirates,” PLoS One 19, no. 1 (2024): e0291373, 10.1371/journal.pone.0291373. 38206939 PMC10783720

[57] L. A. Amar , A. A. Taha , and M. Y. Mohamed , “Prediction of the Final Size for COVID‐19 Epidemic Using Machine Learning: A Case Study of Egypt,” Infectious Disease Modelling 5 (2020): 622–634, 10.1016/j.idm.2020.08.008. 32864516 PMC7446670

[58] S. M. Ayyoubzadeh , S. M. Ayyoubzadeh , H. Zahedi , M. Ahmadi , and S. R Niakan Kalhori , “Predicting COVID‐19 Incidence Through Analysis of Google Trends Data in Iran: Data Mining and Deep Learning Pilot Study,” JMIR Public Health and Surveillance 6, no. 2 (2020): e18828, 10.2196/18828. 32234709 PMC7159058

[59] S. Bashirian , M. Mohammadi‐Khoshnoud , S. Khazaei , et al., “Identification of Risk Factors for COVID‐19‐related Death Using Machine Learning Methods,” Tanaffos 21, no. 1 (2022): 54–62. 36258910 PMC9571237

[60] M. Bodaghie , F. Mahan , L. Sahebi , and H. Dalili , “Neo‐Epidemiological Machine Learning Based Method for COVID‐19 Related Estimations,” PLoS One 18, no. 3 (2023): e0263991, 10.1371/journal.pone.0263991. 36961771 PMC10038265

[61] F. Borhani , M. Shafiepour Motlagh , Y. Rashidi , and A. H. Ehsani , “Estimation of Short‐Lived Climate Forced Sulfur Dioxide in Tehran, Iran, Using Machine Learning Analysis,” Stochastic Environmental Research and Risk Assessment 36, no. 9 (2022): 2847–2860, 10.1007/s00477-021-02167-x. 35035281 PMC8741550

[62] K. K. A. Ghany , H. M. Zawbaa , and H. M. Sabri , “COVID‐19 Prediction Using Lstm Algorithm: GCC Case Study,” Informatics in Medicine Unlocked 23 (2021): 100566, 10.1016/j.imu.2021.100566. 33842686 PMC8021451

[63] K. Hameed Abdulkareem , A. Awad Mutlag , A. Musa Dinar , et al., “Smart Healthcare System for Severity Prediction and Critical Tasks Management of COVID‐19 Patients in IoT‐Fog Computing Environments,” Computational intelligence and neuroscience 2022 (2022): 5012962, 10.1155/2022/5012962. 35875731 PMC9297127

[64] W. Huang , S. Ao , D. Han , Y. Liu , S. Liu , and Y. Huang , “Data‐Driven and Machine‐Learning Methods to Project Coronavirus Disease 2019 Pandemic Trend in Eastern Mediterranean,” Frontiers in Public Health 9 (2021): 602353, 10.3389/fpubh.2021.602353. 34055708 PMC8158576

[65] F. Khounraz , M. Khodadoost , S. Gholamzadeh , et al., “Prognosis of COVID‐19 Patients Using Lab Tests: A Data Mining Approach,” Health Science Reports 6, no. 1 (2023): e1049, 10.1002/hsr2.1049. 36628109 PMC9826741

[66] M. Marzouk , N. Elshaboury , A. Abdel‐Latif , and S. Azab , “Deep Learning Model for Forecasting COVID‐19 Outbreak in Egypt,” Process Safety and Environmental Protection 153 (2021): 363–375, 10.1016/j.psep.2021.07.034. 34334966 PMC8305306

[67] A. I. Saba and A. H. Elsheikh , “Forecasting the Prevalence of COVID‐19 Outbreak in Egypt Using Nonlinear Autoregressive Artificial Neural Networks,” Process Safety and Environmental Protection 141 (2020): 1–8, 10.1016/j.psep.2020.05.029. 32501368 PMC7237379

[68] A. Sharifi‐Kia , A. Nahvijou , and A. Sheikhtaheri , “Machine Learning‐Based Mortality Prediction Models for Smoker COVID‐19 Patients,” BMC Medical Informatics and Decision Making 23, no. 1 (2023): 129, 10.1186/s12911-023-02237-w. 37479990 PMC10360290

[69] S. Moslehi , N. Rabiei , A. R. Soltanian , and M. Mamani , “Application of Machine Learning Models Based on Decision Trees in Classifying the Factors Affecting Mortality of COVID‐19 Patients in Hamadan, Iran,” BMC Medical Informatics and Decision Making 22, no. 1 (2022): 192, 10.1186/s12911-022-01939-x. 35871639 PMC9308952

[70] S. V. Razavi‐Termeh , A. Sadeghi‐Niaraki , F. Farhangi , and S. M. Choi , “COVID‐19 Risk Mapping With Considering Socio‐Economic Criteria Using Machine Learning Algorithms,” International Journal of Environmental Research and Public Health 18, no. 18 (2021): 9657, 10.3390/ijerph18189657. 34574582 PMC8471719

[71] M. J. Shaibani , S. Emamgholipour , and S. S. Moazeni , “Investigation of Robustness of Hybrid Artificial Neural Network With Artificial Bee Colony and Firefly Algorithm in Predicting COVID‐19 New Cases: Case Study of Iran,” Stochastic Environmental Research and Risk Assessment 36, no. 9 (2022): 2461–2476, 10.1007/s00477-021-02098-7. 34608374 PMC8481113

[72] N. Amoori , B. Cheraghian , P. Amini , and S. M. Alavi , “Identification of Risk Factors Associated With Tuberculosis in Southwest Iran: A Machine Learning Method,” Medical Journal of the Islamic Republic of Iran 38 (2024): 5, 10.47176/mjiri.38.5. 38434222 PMC10907055

[73] H. Bagheri , L. Tapak , M. Karami , et al., “Forecasting the Monthly Incidence Rate of Brucellosis in West of Iran Using Time Series and Data Mining From 2010 to 2019,” PLoS One 15, no. 5 (2020): e0232910, 10.1371/journal.pone.0232910. 32396582 PMC7217463

[74] M. Sallam , K. Al‐Salahat , and E. Al‐Ajlouni , “Chatgpt Performance in Diagnostic Clinical Microbiology Laboratory‐Oriented Case Scenarios,” Cureus 15, no. 12 (2023): e50629, 10.7759/cureus.50629. 38107211 PMC10725273

[75] M. R. Saybani , S. Shamshirband , S. Golzari Hormozi , et al., “Diagnosing Tuberculosis With a Novel Support Vector Machine‐Based Artificial Immune Recognition System,” Iranian Red Crescent Medical Journal 17, no. 4 (2015): e24557, 10.5812/ircmj.17(4)2015.24557. 26023340 PMC4443397

[76] M. R. Saybani , S. Shamshirband , S. Golzari , et al., “RAIRS2 a New Expert System for Diagnosing Tuberculosis With Real‐World Tournament Selection Mechanism Inside Artificial Immune Recognition System,” Medical & Biological Engineering & Computing 54, no. 2–3 (2016): 385–399, 10.1007/s11517-015-1323-6. 26081904

[77] N. Shirmohammadi‐Khorram , L. Tapak , O. Hamidi , and Z. Maryanaji , “A Comparison of Three Data Mining Time Series Models in Prediction of Monthly Brucellosis Surveillance Data,” Zoonoses and Public Health 66, no. 7 (2019): 759–772, 10.1111/zph.12622. 31305019

[78] L. A. AlKaabi , L. S. Ahmed , M. F. Al Attiyah , and M. E. Abdel‐Rahman , “Predicting Hypertension Using Machine Learning: Findings From Qatar Biobank Study,” PLoS One 15, no. 10 (2020): e0240370, 10.1371/journal.pone.0240370. 33064740 PMC7567367

[79] F. Alsayegh , M. A. Alkhamis , F. Ali , S. Attur , N. M. Fountain‐Jones , and M. Zubaid , “Anemia or Other Comorbidities? Using Machine Learning to Reveal Deeper Insights into the Drivers of Acute Coronary Syndromes in Hospital Admitted Patients,” PLoS One 17, no. 1 (2022): e0262997, 10.1371/journal.pone.0262997. 35073375 PMC8786175

[80] İ. E. Emre , N. Erol , Y. İ. Ayhan , Y. Özkan , and Ç. Erol , “The Analysis of the Effects of Acute Rheumatic Fever in Childhood on Cardiac Disease With Data Mining,” International Journal of Medical Informatics 123 (2019): 68–75, 10.1016/j.ijmedinf.2018.12.009. 30654905

[81] A. AlShareedah , H. Zidoum , S. Al‐Sawafi , B. Al‐Lawati , and A. Al‐Ansari , “Machine Learning Approach for Predicting Systemic Lupus Erythematosus in Oman‐Based Cohort,” Sultan Qaboos University Medical Journal 23, no. 3 (2023): 328–335, 10.18295/squmj.12.2022.069. 37655084 PMC10467556

[82] R. Hassanzadeh , M. Farhadian , and H. Rafieemehr , “Hospital Mortality Prediction in Traumatic Injuries Patients: Comparing Different Smote‐Based Machine Learning Algorithms,” BMC Medical Research Methodology 23, no. 1 (2023): 101, 10.1186/s12874-023-01920-w. 37087425 PMC10122327

[83] M. Nourelahi , F. Dadboud , H. Khalili , A. Niakan , and H. Parsaei , “A Machine Learning Model for Predicting Favorable Outcome in Severe Traumatic Brain Injury Patients After 6 Months,” Acute and Critical Care 37, no. 1 (2022): 45–52, 10.4266/acc.2021.00486. 34762793 PMC8918709

[84] M. John and H. Shaiba , “Main Factors Influencing Recovery in MERS Co‐V Patients Using Machine Learning,” Journal of Infection and Public Health 12, no. 5 (2019): 700–704, 10.1016/j.jiph.2019.03.020. 30979679 PMC7102802

[85] S. V. Razavi‐Termeh , A. Sadeghi‐Niaraki , and S. M. Choi , “Effects of Air Pollution in Spatio‐Temporal Modeling of Asthma‐Prone Areas Using a Machine Learning Model,” Environmental Research 200 (2021): 111344, 10.1016/j.envres.2021.111344. 34015292

[86] N. Sancar and S. S. Tabrizi , “Machine Learning Approach for the Detection of Vitamin D Level: A Comparative Study,” BMC Medical Informatics and Decision Making 23, no. 1 (2023): 219, 10.1186/s12911-023-02323-z. 37845674 PMC10580577

[87] G. Turk , M. Ozdemir , R. Zeydan , Y. Turk , Z. Bilgin , and E. Zeydan , “On the Identification of Thyroid Nodules Using Semi‐Supervised Deep Learning,” International Journal for Numerical Methods in Biomedical Engineering 37, no. 3 (2021): e3433, 10.1002/cnm.3433. 33389785

[88] P. ForouzeshFar , A. A. Safaei , F. Ghaderi , and S. S. Hashemikamangar , “Dental Caries Diagnosis From Bitewing Images Using Convolutional Neural Networks,” BMC Oral health 24, no. 1 (2024): 211, 10.1186/s12903-024-03973-9. 38341526 PMC10858561

[89] K. Giannakopoulos , A. Kavadella , A. Aaqel Salim , V. Stamatopoulos , and E. G. Kaklamanos , “Evaluation of the Performance of Generative AI Large Language Models ChatGPT, Google Bard, and Microsoft Bing Chat in Supporting Evidence‐Based Dentistry: Comparative Mixed Methods Study,” Journal of Medical Internet Research 25 (2023): e51580, 10.2196/51580. 38009003 PMC10784979

[90] G. Keser , İ. Ş. Bayrakdar , F. N. Pekiner , Ö. Çelik , and K. Orhan , “A Deep Learning Algorithm for Classification of Oral Lichen Planus Lesions From Photographic Images: A Retrospective Study,” Journal of stomatology, oral and maxillofacial surgery 124, no. 1 (2023): 101264, 10.1016/j.jormas.2022.08.007. 35964938

[91] O. Demiray , E. D. Gunes , E. Kulak , et al., “Classification of Patients With Chronic Disease by Activation Level Using Machine Learning Methods,” Health Care Management Science 26, no. 4 (2023): 626–650, 10.1007/s10729-023-09653-4. 37824033

[92] M. Habib , M. Faris , R. Qaddoura , M. Alomari , A. Alomari , and H. Faris , “Toward an Automatic Quality Assessment of Voice‐Based Telemedicine Consultations: A Deep Learning Approach,” Sensors 21, no. 9 (2021): 3279, 10.3390/s21093279. 34068602 PMC8126050

[93] S. S. Momahhed , S. E. Sefiddashti , B. Minaei , and M. Arab , “The Optimal Co‐Insurance Rate for Outpatient Drug Expenses of Iranian Health Insured Based on the Data Mining Method,” International Journal for Equity in Health 23, no. 1 (2024): 25, 10.1186/s12939-023-02065-4. 38331790 PMC10854021

[94] M. Soltani , M. Farahmand , and A. R. Pourghaderi , “Machine Learning‐Based Demand Forecasting in Cancer Palliative Care Home Hospitalization,” Journal of Biomedical Informatics 130 (2022): 104075, 10.1016/j.jbi.2022.104075. 35490963

[95] E. Koç and M. Türkoğlu , “Forecasting of Medical Equipment Demand and Outbreak Spreading Based on Deep Long Short‐Term Memory Network: The COVID‐19 Pandemic in Turkey,” Signal, Image and Video Processing 16, no. 3 (2022): 613–621, 10.1007/s11760-020-01847-5. 33520001 PMC7829095

[96] A. M. Rashed , N. E. El‐Attar , D. S. Abdelminaam , and M. Abdelfatah , “Analysis the Patients' Careflows Using Process Mining,” PLoS One 18, no. 2 (2023): e0281836, 10.1371/journal.pone.0281836. 36821535 PMC9949667

[97] T. Ahmed Ali. Torad , E. Fayiz el‐Shamy , T. Ahmed Mahmoud Tayee , and A. Zeinab Sami Ahmed , “Using Machine Learning Models to Investigate the Relationship Between Corporeal Workload and Clinical and Epidemiological Features of Patients Infected With COVID‐19 In Egypt,” Journal of the Pakistan Medical Association 73, no. 4 (2023): S242–S246, 10.47391/jpma.Egy-s4-48. 37482866

[98] A. Naghavi , T. Teismann , Z. Asgari , M. R. Mohebbian , M. Mansourian , and M. Á. Mañanas , “Accurate Diagnosis of Suicide Ideation/Behavior Using Robust Ensemble Machine Learning: A University Student Population in the Middle East and North Africa (MENA) Region,” Diagnostics (Basel) 10, no. 11 (November 2020): 956, 10.3390/diagnostics10110956. 33207776 PMC7696788

[99] R. Qasrawi , M. Amro , S. VicunaPolo , et al., “Machine Learning Techniques for Predicting Depression and Anxiety in Pregnant and Postpartum Women During the COVID‐19 Pandemic: A Cross‐Sectional Regional Study,” F1000Research 11 (2022): 390, 10.12688/f1000research.110090.1. 36111217 PMC9445566

[100] R. Qasrawi , S. Vicuna Polo , R. Abu Khader , et al., “Machine Learning Techniques for Identifying Mental Health Risk Factor Associated With Schoolchildren Cognitive Ability Living in Politically Violent Environments,” Frontiers in Psychiatry 14 (2023): 1071622, 10.3389/fpsyt.2023.1071622. 37304448 PMC10250653

[101] M. Seyedtabib and N. Kamyari , “Predicting Polypharmacy in Half a Million Adults in the Iranian Population: Comparison of Machine Learning Algorithms,” BMC Medical Informatics and Decision Making 23, no. 1 (2023): 84, 10.1186/s12911-023-02177-5. 37147615 PMC10161984

[102] R. Alshalan , H. Al‐Khalifa , D. Alsaeed , H. Al‐Baity , and S. Alshalan , “Detection of Hate Speech in COVID‐19‐Related Tweets in the Arab Region: Deep Learning and Topic Modeling Approach,” Journal of Medical Internet Research 22, no. 12 (2020): e22609, 10.2196/22609. 33207310 PMC7725497

[103] G. Dikeç , V. Oban , and M. Barış Usta , “Qualitative and Artificial Intelligence‐Based Sentiment Analysis of Turkish Tweets Related to Schizophrenia,” Turk psikiyatri dergisi = Turkish Journal of Psychiatry 34, no. 3 (2023): 145–153, 10.5080/u26402. 37724640 PMC10645022

[104] P. Göksel , V. Oban , G. Dikeç , and M. B. Usta , “Qualitative and Artificial Intelligence‐Based Sentiment Analysis of Turkish Twitter Messages Related to Autism Spectrum Disorders,” Cureus 15, no. 5 (2023): e38446, 10.7759/cureus.38446. 37143854 PMC10153655

[105] M. Salehi , S. Ghahari , M. Hosseinzadeh , and L. Ghalichi , “Domestic Violence Risk Prediction in Iran Using a Machine Learning Approach by Analyzing Persian Textual Content in Social Media,” Heliyon 9, no. 5 (2023): e15667, 10.1016/j.heliyon.2023.e15667. 37180917 PMC10172903

[106] R. Qasrawi , M. Hoteit , R. Tayyem , et al., “Machine Learning Techniques for the Identification of Risk Factors Associated With Food Insecurity Among Adults in Arab Countries During the COVID‐19 Pandemic,” BMC Public Health 23, no. 1 (2023): 1805, 10.1186/s12889-023-16694-5. 37716999 PMC10505318

[107] A. AlShehhi and R. Welsch , “Artificial Intelligence for Improving Nitrogen Dioxide Forecasting of Abu Dhabi Environment Agency Ground‐Based Stations,” Journal of Big Data 10, no. 1 (2023): 92, 10.1186/s40537-023-00754-z. 37303479 PMC10236404

[108] H. Mahboobi , A. Shakiba , and B. Mirbagheri , “Improving Groundwater Nitrate Concentration Prediction Using Local Ensemble of Machine Learning Models,” Journal of Environmental Management 345 (2023): 118782, 10.1016/j.jenvman.2023.118782. 37597371

[109] A. Al Yammahi and Z. Aung , “Forecasting the Concentration of NO2 Using Statistical and Machine Learning Methods: A Case Study in the UAE,” Heliyon 9, no. 2 (2023): e12584, 10.1016/j.heliyon.2022.e12584. 36793966 PMC9922785

[110] H. Gholami , A. Mohammadifar , R. D. Behrooz , D. G. Kaskaoutis , Y. Li , and Y. Song , “Intrinsic and Extrinsic Techniques for Quantification Uncertainty of an Interpretable GRU Deep Learning Model Used to Predict Atmospheric Total Suspended Particulates (TSP) in Zabol, Iran During the Dusty Period of 120‐days Wind,” Environmental Pollution 342 (2024): 123082, 10.1016/j.envpol.2023.123082. 38061429

[111] J. Li , E. Garshick , J. E. Hart , et al., “Estimation of Ambient PM(2.5) in Iraq and Kuwait From 2001 to 2018 Using Machine Learning and Remote Sensing,” Environment International 151 (2021): 106445, 10.1016/j.envint.2021.106445. 33618328 PMC8023768

[112] J. Li , C. M. Kang , J. M. Wolfson , et al., “Estimation of Fine Particulate Matter in an Arid Area From Visibility Based on Machine Learning,” Journal of Exposure Science & Environmental Epidemiology 32, no. 6 (2022): 926–931, 10.1038/s41370-022-00480-3. 36151455 PMC9742157

[113] S. Z. Shogrkhodaei , S. V. Razavi‐Termeh , and A. Fathnia , “Spatio‐Temporal Modeling of PM(2.5) Risk Mapping Using Three Machine Learning Algorithms,” Environmental Pollution 289 (2021): 117859, 10.1016/j.envpol.2021.117859. 34340183

[114] H. Tao , A. H. Jawad , A. H. Shather , et al., “Machine Learning Algorithms for High‐Resolution Prediction of Spatiotemporal Distribution of Air Pollution From Meteorological and Soil Parameters,” Environment International 175 (2023): 107931, 10.1016/j.envint.2023.107931. 37119651

[115] E. Günal , M. Budak , M. Kılıç , B. Cemek , and M. Sırrı , “Combining Spatial Autocorrelation With Artificial Intelligence Models to Estimate Spatial Distribution and Risks of Heavy Metal Pollution in Agricultural Soils,” Environmental Monitoring and Assessment 195, no. 2 (2023): 317, 10.1007/s10661-022-10813-2. 36680597

[116] M. Jamei , I. Ahmadianfar , M. Karbasi , A. H. Jawad , A. A. Farooque , and Z. M. Yaseen , “The Assessment of Emerging Data‐Intelligence Technologies for Modeling Mg(+2) and SO(4)(‐2) Surface Water Quality,” Journal of Environmental Management 300 (2021): 113774, 10.1016/j.jenvman.2021.113774. 34560461

[117] A. Mohammadpour , M. Keshtkar , M. R. Samaei , S. Isazadeh , and A. Mousavi Khaneghah , “Assessing Water Quality Index and Health Risk Using Deterministic and Probabilistic Approaches in Darab County, Iran; A Machine Learning for Fluoride Prediction,” Chemosphere 352 (2024): 141284, 10.1016/j.chemosphere.2024.141284. 38336038

[118] S. V. Razavi‐Termeh , A. Sadeghi‐Niaraki , R. A. Naqvi , and S. M. Choi , “Dust Detection and Susceptibility Mapping by Aiding Satellite Imagery Time Series and Integration of Ensemble Machine Learning With Evolutionary Algorithms,” Environmental Pollution 335 (2023): 122241, 10.1016/j.envpol.2023.122241. 37482338

[119] T. Zhang , Y. Li , and M. Wang , “Remote Sensing‐Based Prediction of Organic Carbon in Agricultural and Natural Soils Influenced by Salt and Sand Mining Using Machine Learning,” Journal of Environmental Management 352 (2024): 120107, 10.1016/j.jenvman.2024.120107. 38237334

[120] D. Hammoudi Halat , A. S. G. Abdel‐Salam , A. Bensaid , et al., “Use of Machine Learning to Assess Factors Affecting Progression, Retention, and Graduation in First‐Year Health Professions Students in Qatar: A Longitudinal Study,” BMC Medical Education 23, no. 1 (2023): 909, 10.1186/s12909-023-04887-w. 38036997 PMC10691082

[121] M. C. E. Simsekler , N. H. Alhashmi , E. Azar , N. King , R. A. M. A. Luqman , and A. Al Mulla , “Exploring Drivers of Patient Satisfaction Using a Random Forest Algorithm,” BMC Medical Informatics and Decision Making 21, no. 1 (2021): 157, 10.1186/s12911-021-01519-5. 33985481 PMC8120836

[122] Z. Hamedani , M. Moradi , F. Kalroozi , et al., “Evaluation of Acceptance, Attitude, and Knowledge Towards Artificial Intelligence and Its Application From the Point of View of Physicians and Nurses: A Provincial Survey Study in Iran: A Cross‐Sectional Descriptive‐Analytical Study,” Health Science Reports 6, no. 9 (2023): e1543, 10.1002/hsr2.1543. 37674620 PMC10477406

[123] P. Shojaei , M. Khosravi , Y. Jafari , A. H. Mahmoudi , and H. Hassanipourmahani , “Chatgpt Utilization Within the Building Blocks of the Healthcare Services: A Mixed‐Methods Study,” Digital Health 10 (2024): 20552076241297059, 10.1177/20552076241297059. 39559384 PMC11571260

[124] A. Al Kuwaiti , K. Nazer , A. Al‐Reedy , et al., “A Review of the Role of Artificial Intelligence in Healthcare,” Journal of Personalized Medicine 13, no. 6 (2023): 951, 10.3390/jpm13060951. 37373940 PMC10301994

[125] S. A. Alowais , S. S. Alghamdi , N. Alsuhebany , et al., “Revolutionizing Healthcare: The Role of Artificial Intelligence in Clinical Practice,” BMC Medical Education 23, no. 1 (2023): 689, 10.1186/s12909-023-04698-z. 37740191 PMC10517477

[126] F. Farhat , S. S. Sohail , M. T. Alam , et al., “COVID‐19 and Beyond: Leveraging Artificial Intelligence for Enhanced Outbreak Control,” Frontiers in Artificial Intelligence 6 (2023): 1266560, 10.3389/frai.2023.1266560. 38028660 PMC10663297

[127] F. Piccialli , V. S. di Cola , F. Giampaolo , and S. Cuomo , “The Role of Artificial Intelligence in Fighting the COVID‐19 Pandemic,” Information Systems Frontiers 23, no. 6 (2021): 1467–1497, 10.1007/s10796-021-10131-x. 33935585 PMC8072097

[128] R. Vaishya , M. Javaid , I. H. Khan , and A. Haleem , “Artificial Intelligence (AI) Applications for COVID‐19 Pandemic,” Diabetes & Metabolic Syndrome: Clinical Research & Reviews 14, no. 4 (2020): 337–339, 10.1016/j.dsx.2020.04.012. PMC7195043 32305024

[129] Z. Chang , Z. Zhan , Z. Zhao , et al., “Application of Artificial Intelligence in COVID‐19 Medical Area: A Systematic Review,” Journal of Thoracic Disease 13, no. 12 (2021): 7034–7053, 10.21037/jtd-21-747. 35070385 PMC8743418

[130] M. Ahmadi Marzaleh , M. Peyravi , S. Mousavi , F. Sarpourian , M. Seyedi , and N. Shalyari , “Artificial Intelligence Functionalities During the COVID‐19 Pandemic,” Disaster Medicine and Public Health Preparedness 17 (2023): e336, 10.1017/dmp.2023.3. 36847255

[131] A. Znaor , S. Eser , K. Bendahhou , et al., “Stage at Diagnosis of Colorectal Cancer in the Middle East and Northern Africa: A Population‐Based Cancer Registry Study,” International Journal of Cancer 155 (2024): 54–60, 10.1002/ijc.34895. 38456478

[132] B. Abuyassin and I. Laher , “Diabetes Epidemic Sweeping the Arab World,” World Journal of Diabetes 7, no. 8 (2016): 165–174, 10.4239/wjd.v7.i8.165. 27114755 PMC4835661

[133] I. M. El‐Kebbi , N. H. Bidikian , L. Hneiny , and M. P. Nasrallah , “Epidemiology of Type 2 Diabetes in the Middle East and North Africa: Challenges and Call for Action,” World Journal of Diabetes 12, no. 9 (2021): 1401–1425, 10.4239/wjd.v12.i9.1401. 34630897 PMC8472500

[134] N. Al Busaidi , P. Shanmugam , and D. Manoharan , “Diabetes in the Middle East: Government Health Care Policies and Strategies That Address the Growing Diabetes Prevalence in the Middle East,” Current diabetes reports 19, no. 2 (2019): 8, 10.1007/s11892-019-1125-6. 30715611

[135] A. Robert , M. Al Dawish , R. Braham , M. Musallam , A. Al Hayek , and N. Al Kahtany , “Type 2 Diabetes Mellitus in Saudi Arabia: Major Challenges and Possible Solutions,” Current Diabetes Reviews 13, no. 1 (2016): 59–64, 10.2174/1573399812666160126142605. 26813972

[136] M. Neira , K. Erguler , H. Ahmady‐Birgani , et al., “Climate Change and Human Health in the Eastern Mediterranean and Middle East: Literature Review, Research Priorities and Policy Suggestions,” Environmental Research 216, no. Pt 2 (2023): 114537, 10.1016/j.envres.2022.114537. 36273599 PMC9729515

[137] S. Paz , A. Majeed , and G. K. Christophides , “Climate Change Impacts on Infectious Diseases in the Eastern Mediterranean and the Middle East (EMME)‐Risks and Recommendations,” Climatic Change 169, no. 3–4 (2021): 40, 10.1007/s10584-021-03300-z. 34980932 PMC8716574

