# Supplementary material for: A Systematic Review of the Outcomes of Utilization of Artificial Intelligence Within the Healthcare Systems of the Middle East: A Thematic Analysis of Findings
Source: Health Sci Rep. 2024 Dec 24;7(12):e70300. doi: 10.1002/hsr2.70300 (PMC11667773; doi:10.1002/hsr2.70300)
Supplement: Supplementary file 1 — Supporting information. [file HSR2-7-e70300-s001.docx]

**Appendix 1. Bibliography of final studies.**

| **No.** | **Year** | **Country** | **Context** | **Study type** | **AI type** | **Summary** | **Reference** |
| --- | --- | --- | --- | --- | --- | --- | --- |
| 1 | 2021 | Oman | Physical disorders | Quantitative | Machine learning | By accurately identifying individuals at risk of insulin resistance at an early stage, this method may help prevent or delay the onset of chronic diseases like type 2 diabetes. This highlights the potential clinical impact of utilizing AI in healthcare for early disease detection and intervention. | (1) |
| 2 | 2022 | Iran | Physical disorders | Quantitative | Machine learning | AI, specifically a genetic algorithm (GA) incorporating multiple machine learning (ML) algorithms, was employed to develop a predictive model for breast cancer (BC) prediction and early warning.  Based on the best-performing model (decision tree), a clinical decision support system was developed. This system can accurately identify individuals at elevated risk for BC, serving as an important screening tool for early prevention and aiding in the development of preventive health strategies. | (2) |
| 3 | 2018 | Turkey | Physical disorders | Quantitative | Machine learning | AI techniques were used to develop a prediction system with assistive e-Health applications. This system aimed to aid both pregnant women and practitioners in predicting fetal congenital anomalies beyond traditional pregnancy tests. | (3) |
| 4 | 2023 | Oman | Physical disorders | Quantitative | Machine learning | The study developed a novel 4D CNN model dedicated to early diabetes prediction. This model leverages machine learning techniques to analyze medical data and identify individuals at risk of developing diabetes. | (4) |
| 5 | 2023 | UAE | Environmental issues | Quantitative | Machine learning | AI techniques, including statistical and machine learning models such as autoregressive integrated moving average (ARIMA), seasonal autoregressive integrated moving average (SARIMA), long short-term memory (LSTM), and nonlinear autoregressive neural network (NAR-NN), were employed to predict the concentration of nitrogen dioxide (NO2) at 14 ground stations in the United Arab Emirates (UAE) during December 2020. | (5) |
| 6 | 2018 | Palestine | Physical disorders | Quantitative | Machine learning | The dataset is used to predict the specific type of thalassemia known as beta thalassemia (β-thalassemia) using a hybrid data mining model. | (6) |
| 7 | 2023 | Saudi Arabia | Physical disorders | Quantitative | Machine learning | Statistical and machine learning methods were used to predict factors associated with multimorbidity in the Saudi population. The study aimed to identify common modifiable behavioral risk factors that contribute to multimorbidity. | (7) |
| 8 | 2020 | Qatar | Physical disorders | Quantitative | Machine learning | The study aimed to construct predictive models to identify individuals at high risk of developing hypertension using non-invasive predictors. Three supervised machine learning algorithms were utilized: decision tree, random forest, and logistic regression. | (8) |
| 9 | 2022 | Saudi Arabia | Physical disorders | Quantitative | Machine learning | The study describes the experience of a large tertiary hospital system network in the Middle East in developing a real-time severity prediction tool for COVID-19 patients. This tool aims to assist clinicians in matching patients with appropriate levels of needed care for better management of limited healthcare resources during COVID-19 surges. | (9) |
| 10 | 2022 | Bahrain, Kuwait, Oman, UAE | Physical disorders | Quantitative | Machine learning | The study aimed to uncover deeper insights into the factors that shape acute coronary syndromes (ACS) outcomes in hospitals across four Arabian Gulf countries. AI techniques were employed to analyze and understand the complex relationships between ACS and patient risk factors. | (10) |
| 11 | 2020 | Saudi Arabia | Social disorders | Quantitative | Machine learning | The study aimed to automatically detect hate speech related to the COVID-19 pandemic posted by Twitter users in the Arab region. A pretrained CNN model was used to analyze tweets, assigning each tweet a score between 0 and 1, with 1 indicating the most hateful text. | (11) |
| 12 | 2023 | Oman | Physical disorders | Quantitative | Machine learning | The study aimed to design a machine learning-based prediction framework to predict the presence or absence of systemic lupus erythematosus (SLE) in a cohort of Omani patients. | (12) |
| 13 | 2024 | UAE | Physical disorders | Quantitative | Machine learning | The study aimed to evaluate the clinical characteristics and risk factors associated with COVID-19 patients in the United Arab Emirates. ML models were used for survival analysis, which involved assessing the likelihood of survival over time based on various clinical parameters. | (13) |
| 14 | 2023 | UAE | Environmental issues | Quantitative | Machine learning | The study compares the performance of several state-of-the-art artificial intelligence models for predicting the concentration of Nitrogen Dioxide (NO2). These models include LSTM, InceptionTime, ResNet, XceptionTime, MiniRocket, and a transformer model. | (14) |
| 15 | 2020 | Egypt | Physical disorders | Quantitative | Machine learning | The study analyzes the real database of COVID-19 for Egypt from February 15, 2020, to June 15, 2020. It aims to predict the number of patients that will be infected with COVID-19 and estimate the final size of the epidemic. | (15) |
| 16 | 2024 | Iran | Physical disorders | Quantitative | Machine learning | The research aims to create a diagnostic aid system for tuberculosis screening using machine learning techniques. This system assists in the early diagnosis of tuberculosis by identifying economic, social, and environmental factors associated with the disease. | (16) |
| 17 | 2018 | Iran | Physical disorders | Quantitative | Machine learning | A data-mining algorithm was developed and implemented using R software to analyze claims data from the Iran Social Security Organization. This algorithm helped identify HER2-positive breast cancer patients and classify them into three main stages: early, loco-regional, and advanced. | (17) |
| 18 | 2020 | Iran | Physical disorders | Quantitative | Machine learning | The use of data mining algorithms, such as linear regression and LSTM, can aid in predicting trends of disease outbreaks like COVID-19. | (18) |
| 19 | 2020 | Iran | Physical disorders | Quantitative | Machine learning | The study aimed to assess and compare the performance of four machine-learning methods in modeling and forecasting brucellosis time series data based on climatic parameters. The results indicate that the multilayer perceptron neural network demonstrated better performance in forecasting the data compared to other models. This suggests that AI, specifically in the form of neural networks, can improve the accuracy of disease outbreak forecasts. | (19) |
| 20 | 2022 | Iran | Physical disorders | Quantitative | Machine learning | AI techniques, including logistic regression, decision tree, and random forest, were employed to identify risk factors associated with mortality due to COVID-19 in Hamadan, Iran. These techniques allowed for the analysis of various medical and demographic factors to determine their impact on the likelihood of death from the disease. | (20) |
| 21 | 2023 | Iran | Physical disorders | Quantitative | Machine learning | The study aimed to estimate the rates of morbidity, growth, and mortality for COVID-19 over a three-month period in Iran. AI techniques were used to analyze data obtained from various sources including daily reports from the World Health Organization (WHO), Iran Meteorological Organization (IRIMO), and the Statistics Center of Iran. | (21) |
| 22 | 2022 | Iran | Physical disorders | Quantitative | Machine learning | The utilization of AI in the study enabled the systematic analysis and estimation of key epidemiological parameters related to COVID-19, including morbidity, growth, and mortality rates. These outcomes contribute to a better understanding of the pandemic's trajectory and facilitate evidence-based decision-making for public health authorities and policymakers. | (22) |
| 23 | 2021 | Turkey | Physical disorders | Quantitative | Machine learning | The utilization of AI algorithms in the study facilitated the identification of optimal models for early diagnosis and prognosis of Malignant Pleural Mesothelioma, providing valuable insights for improving patient care and healthcare resource allocation. | (23) |
| 24 | 2023 | Iran | Physical disorders | Quantitative | Machine learning | The utilization of AI techniques in the study provided valuable insights into the predicting factors for delayed breast cancer diagnosis in women in Iran, informing potential strategies for improving early detection and reducing mortality rates associated with BC. | (24) |
| 25 | 2023 | Turkey | Physical disorders | Quantitative | Machine learning | The utilization of AI techniques in the study provided valuable insights into the factors influencing patient activation levels and demonstrated the effectiveness of Logistic Regression in classifying patients based on their PAM levels. | (25) |
| 26 | 2023 | Iran | Physical disorders | Quantitative | Machine learning | The utilization of AI techniques in the study provided valuable insights into the risk factors for primary invasive incident breast cancer in the Iranian population, facilitating improved BC prevention and management strategies tailored to individual risk profiles. | (26) |
| 27 | 2023 | Turkey | Mental disorders | Quantitative | Machine learning | The utilization of AI for emotional analysis of Turkish tweets about schizophrenia provided insights into the prevalence of negative emotions and stigmatizing attitudes in social media discourse, highlighting the importance of addressing stigma and discrimination in mental health communication and advocacy efforts. | (27) |
| 28 | 2022 | Turkey | Physical disorders | Quantitative | Machine learning | The majority of respondents, 70.3%, identified 'rapid diagnosis' as the most important advantage of AI in breast healthcare. This suggests that AI can potentially expedite the diagnostic process, leading to quicker treatment decisions. | (28) |
| 29 | 2019 | Turkey | Physical disorders | Quantitative | Machine learning | The outcomes of utilizing AI through data mining techniques in this study showcase its potential to improve diagnosis, treatment, and resource utilization in healthcare, specifically in the context of ARF and its impact on cardiac diseases. | (29) |
| 30 | 2018 | Iran | Physical disorders | Quantitative | Machine learning | The outcomes highlight the utility of AI models in analyzing complex datasets to identify and predict risk factors associated with type 2 diabetes, thereby providing valuable insights for healthcare professionals and policymakers in managing and preventing this global health burden. | (30) |
| 31 | 2020 | Tehran | Physical disorders | Quantitative | Machine learning | The study demonstrates the potential of machine learning algorithms, particularly decision tree methods, in predicting cervical cancer and identifying relevant predictors, thereby providing valuable insights for healthcare professionals in cervical cancer prevention and management. | (31) |
| 32 | 2019 | Kuwait | Physical disorders | Quantitative | Machine learning | The outcomes highlight the potential of machine-learning algorithms in enhancing the prediction of T2DM risk and facilitating the development of targeted prevention strategies for high-risk individuals in the Arab population. | (32) |
| 33 | 2013 | Kuwait | Physical disorders | Quantitative | Machine learning | The outcomes of utilizing AI in the form of machine-learning algorithms provide valuable tools for predicting the onset of diabetes, hypertension, and comorbidity, highlighting the significance of ethnicity and regional data in risk assessment and early intervention strategies. | (33) |
| 34 | 2024 | Iran | Physical disorders | Quantitative | Machine learning | The outcomes demonstrate the potential of AI, particularly CNNs, in revolutionizing dental diagnosis by providing accurate and efficient tools for detecting dental caries from radiology images, thereby improving patient care and reducing the burden on dental professionals. | (34) |
| 35 | 2022 | Iraq | Physical disorders | Quantitative | Machine learning | The outcomes of utilizing AI techniques in health data analysis offer valuable insights into disease patterns, patient outcomes, and risk factors, ultimately contributing to improved healthcare delivery and public health | (35) |
| 36 | 2021 | Bahrain, Kuwait, Qatar, Saudi Arabia, UAE, Oman | Physical disorders | Quantitative | Machine learning | The outcomes highlight the effectiveness of AI, specifically LSTM algorithms, in predicting the propagation of COVID-19 and providing valuable insights into recovery periods and error metrics for decision-making purposes in the Gulf Cooperation Council countries. | (36) |
| 37 | 2024 | Iran | Environmental issues | Quantitative | Machine learning | The outcomes demonstrate the effectiveness of utilizing AI, particularly GRU deep learning models, for predicting TSP concentrations and highlight the importance of uncertainty quantification and interpretability techniques in enhancing the understanding and trustworthiness of predictive models in environmental science. | (37) |
| 38 | 2023 | Cyprus | Physical disorders | Quantitative | Machine learning | The study concludes that while LLMs hold promise as aids in evidence-based dentistry. | (38) |
| 39 | 2023 | Turkey | Mental disorders | Quantitative | Machine learning | The utilization of Artificial Intelligence in emotion analysis of Twitter messages provides valuable insights into the perceptions, attitudes, and discourse surrounding autism in Turkish society, contributing to a better understanding of the challenges and opportunities for advocacy and support. | (39) |
| 40 | 2023 | Turkey | Environmental issues | Quantitative | Machine learning | The utilization of Artificial Intelligence in this study facilitated the assessment of heavy metal pollution in agricultural lands, providing valuable insights into spatial distribution patterns and pollution levels, which can inform decision-making processes and environmental management efforts. | (40) |
| 41 | 2021 | Middle East | Digital health | Quantitative | Machine learning | The use of a deep-learning-based classification model allows for the automation of the quality assessment process of patient-doctor voice-based conversations in a telehealth service. This eliminates the need for manual evaluation by trained experts, making the assessment process more efficient and practical, especially considering the rapid increase in consultations. | (41) |
| 42 | 2023 | Iran | Physical disorders | Quantitative | Machine learning | The utilization of ML-based models demonstrates promising results in accurately predicting the need for neurosurgical intervention in patients with moderate TBIs. These models have the potential to enhance clinical decision-making and optimize patient care in the context of traumatic brain injuries. | (42) |
| 43 | 2022 | Iraq | Physical disorders | Quantitative | Machine learning | The utilization of AI-based techniques, including machine learning models and multi-agent systems, offers promising outcomes for improving the management and prediction of COVID-19 severity in healthcare settings. | (43) |
| 44 | 2023 | Qatar | Healthcare education | Quantitative | Machine learning | The utilization of AI-based predictive modeling provides valuable insights into the factors influencing student success in health professions education, enabling institutions to implement targeted strategies to support student outcomes effectively. | (44) |
| 45 | 2023 | Iran | Physical disorders | Quantitative | Machine learning | The utilization of Artificial Intelligence, particularly SMOTE-based machine learning algorithms, shows promise in predicting hospital mortality in trauma patients and can have significant clinical implications for improving patient care in intensive care settings. | (45) |
| 46 | 2022 | Iran | Physical disorders | Quantitative | Machine learning | The utilization of Artificial Intelligence, particularly machine learning algorithms, facilitated the prediction of diabetic nephropathy incidence in T2DM patients by identifying important risk factors and constructing predictive models with high accuracy. | (46) |
| 47 | 2021 | Qatar, Egypt, Pakistan, Saudi Arabia | Physical disorders | Quantitative | Machine learning | The utilization of AI facilitates the analysis, assessment, and projection of COVID-19 trends and intervention outcomes, contributing to efforts aimed at preventing and controlling the spread of the virus in the Eastern Mediterranean region. | (47) |
| 48 | 2021 | Iran | Environmental issues | Quantitative | Machine learning | The utilization of AI in the form of complementary intelligence paradigms improves the accuracy of forecasting soluble salt concentrations in Maroon River, contributing to better management of drinking water resources and human health in the region. | (48) |
| 49 | 2019 | Saudi Arabia | Physical disorders | Quantitative | Machine learning | The study employs machine learning techniques, including support vector machine, conditional inference tree, naïve Bayes, and J48, to identify the important factors influencing Middle East Respiratory Syndrome (MERS) recovery in the Kingdom of Saudi Arabia (KSA). | (49) |
| 50 | 2020 | Iran | Physical disorders | Quantitative | Machine learning | The study aims to predict the necessity of cranial radiotherapy (CRT) treatment in pediatric acute lymphoblastic leukemia (ALL) patients using machine learning techniques. | (50) |
| 51 | 2023 | Turkey | Physical disorders | Quantitative | Machine learning | The use of AI components in imaging equipment can reduce the effort required by healthcare professionals, increase efficiency, and improve diagnostic capabilities. The preliminary findings of the study suggest that deep learning models have the potential to accurately detect oral lesions, demonstrating the efficacy of AI in medical image analysis. | (51) |
| 52 | 2022 | UAE | Physical disorders | Quantitative | Machine learning | The study demonstrates the effectiveness of AI, particularly ML techniques, in accurately predicting newborn birth weight and classifying infants into LBW and non-LBW categories. These predictive models have the potential to assist healthcare professionals in assessing newborn health and safety, particularly in identifying infants at risk of LBW and its associated health outcomes. | (52) |
| 53 | 2023 | Iran | Physical disorders | Quantitative | Machine learning | The study suggests that data mining methods, particularly ML algorithms, have the potential to predict outcomes of COVID-19 patients using laboratory test results and demographic features. These methods could be implemented in clinical decision support systems to improve the management and care of severe COVID-19 patients after validation. | (53) |
| 54 | 2022 | Turkey | Physical disorders | Quantitative | Machine learning | The experiments demonstrate that the proposed deep learning model can effectively estimate the number of COVID-19 cases and forecast the demand for medical equipment in the future. This suggests that AI-based forecasting models can play a valuable role in managing healthcare resources during infectious disease outbreaks like COVID-19. | (54) |
| 55 | 2021 | Iraq, Kuwait | Environmental issues | Quantitative | Machine learning | This study represents the first attempt to estimate long-term PM(2.5) exposures in Iraq and Kuwait at a high resolution using measurements data. The novel approach demonstrated in this study may be applicable to other regions with limited monitoring networks, providing valuable insights into air quality and its implications for public health. | (55) |
| 56 | 2022 | Iraq, Kuwait | Environmental issues | Quantitative | Machine learning | The ability to retrospectively estimate daily PM(2.5) exposures using historical visibility data in regions with few monitoring stations is significant for environmental risk assessments and population health studies. It provides valuable insights into air quality and its implications for public health in arid regions like Kuwait. | (56) |
| 57 | 2023 | Iran | Environmental issues | Quantitative | Machine learning | By integrating the outputs of ML models using a local approach (geographically weighted regression - GWR), the spatial accuracy of groundwater nitrate concentration prediction is enhanced. This suggests that AI techniques can better capture spatial variation compared to conventional methods like averaging. | (57) |
| 58 | 2021 | Egypt | Physical disorders | Quantitative | Machine learning | The utilization of Artificial Intelligence in predicting the prevalence of the COVID-19 outbreak in Egypt offers significant benefits in terms of improved forecasting accuracy, informed decision-making, and proactive management of the pandemic. | (58) |
| 59 | 2023 | Iran | Physical disorders | Quantitative | Machine learning | Machine learning approaches, such as XGBoost classification, demonstrate high accuracy in predicting postpartum hemorrhage risk. These models outperform traditional analytical approaches, with XGBoost classification being identified as the best-performing model in this study. | (59) |
| 60 | 2024 | Iran | Physical disorders | Quantitative | Machine learning | The utilization of Artificial Intelligence, particularly machine learning methods, facilitates the identification of crucial predictors of CRC patient survival, evaluates model performance, and provides valuable insights for policy recommendations aimed at improving patient outcomes in colorectal cancer treatment and management. | (60) |
| 61 | 2023 | Iran | Physical disorders | Quantitative | Machine learning | The machine learning model identifies several risk factors associated with gastric cancer, including age, social economical status, tea temperature, body mass index, gender, and education. These factors are found to have significant impacts on the occurrence of gastric cancer. | (61) |
| 62 | 2024 | Iran | Environmental issues | Quantitative | Machine learning | The study utilizes a machine-learning algorithm to predict pollution levels in water. This AI-based approach helps in assessing water quality and identifying potential health risks associated with water consumption. | (62) |
| 63 | 2024 | Iran | Organizational issues | Quantitative | Machine learning | The utilization of Artificial Intelligence in estimating optimal co-insurance for outpatient drug costs facilitates more equitable allocation of healthcare funds, improves access to medications for vulnerable populations, and enhances resource efficiency in healthcare systems. | (63) |
| 64 | 2020 | Iran | Physical disorders | Quantitative | Machine learning | By leveraging data mining algorithms, the study aims to improve the accuracy of predicting breast cancer recurrence. By identifying the most relevant features and employing advanced algorithms, such as C5.0, the study contributes to more precise prediction models for better patient management and outcomes.the utilization of Artificial Intelligence, specifically data mining algorithms, in predicting breast cancer recurrence offers promising outcomes in terms of identifying the optimal predictive model, highlighting important predictive features, and improving prediction accuracy. These findings have implications for enhancing clinical decision-making and patient care in the context of breast cancer management. | (64) |
| 65 | 2022 | Iran | Physical disorders | Quantitative | Machine learning | the utilization of Artificial Intelligence, particularly in the form of classification models like CART, offers valuable insights into COVID-19 mortality prediction, aiding healthcare professionals in making informed decisions and prioritizing patient care effectively. | (65) |
| 66 | 2023 | Egypt | Physical disorders | Quantitative | Machine learning | the utilization of Artificial Intelligence, particularly artificial neural networks, and decision tree algorithms, offers a promising approach for predicting diabetic foot ulcers with high accuracy. These predictive models have significant implications for improving patient outcomes and reducing the burden of diabetic complications. | (66) |
| 67 | 2020 | Middle East | Mental disorders | Quantitative | Machine learning | the utilization of Artificial Intelligence in this study provides valuable insights into the identification and prediction of suicide ideation/behavior among university students. These findings can inform targeted interventions and support strategies to address the alarming public health concern of suicide in the MENA region. | (67) |
| 68 | 2023 | Iran | Physical disorders | Quantitative | Machine learning | the utilization of Artificial Intelligence, particularly the random forest model, offers a promising approach to predicting the five-year survival of EC patients. These findings have the potential to inform clinical decision-making and improve the quality of care for EC patients in Iran. | (68) |
| 69 | 2022 | Iran | Physical disorders | Quantitative | Machine learning | the utilization of Artificial Intelligence, particularly machine learning techniques, offers promising capabilities for predicting outcomes in severe TBI patients. These predictive models have significant potential to enhance clinical decision-making and improve patient outcomes in TBI care. | (69) |
| 70 | 2022 | Jordan, Palestine, Lebanon, Saudi Arabia, Bahrain | Mental disorders | Quantitative | Machine learning | the utilization of Artificial Intelligence, particularly ML models, offers promising capabilities for predicting and addressing maternal depression and anxiety during the COVID-19 pandemic, ultimately enhancing maternal and child health outcomes in affected regions. | (70) |
| 71 | 2023 | Jordan, Palestine, Lebanon, Saudi Arabia, Bahrain | Environmental issues | Quantitative | Machine learning | the utilization of Artificial Intelligence, particularly machine learning techniques, offers valuable insights into the dynamics of food insecurity during the COVID-19 pandemic. These insights can inform policymaking efforts aimed at addressing food insecurity and improving access to food among vulnerable communities. | (71) |
| 72 | 2023 | Palestine | Mental disorders | Quantitative | Machine learning | the utilization of Artificial Intelligence, particularly machine learning techniques, enables a comprehensive analysis of the impact of political violence on children's mental health and cognitive development. These insights can inform targeted interventions and policies aimed at improving the well-being of children living in conflict-affected areas. | (72) |
| 73 | 2023 | Iran | Physical disorders | Quantitative | Machine learning | The study utilized a machine learning approach to predict LBW in newborns. LBW is a crucial factor associated with infant mortality and newborn health risks, making its prediction valuable for preventative measures and early intervention. | (73) |
| 74 | 2023 | Egypt | Organizational issues | Quantitative | Machine learning | the utilization of process mining techniques in the healthcare sector in Egypt aims to enhance service delivery, address increasing costs, and improve patient experiences by optimizing careflows and processes. | (74) |
| 75 | 2021 | Iran | Physical disorders | Quantitative | Machine learning | the utilization of machine learning, particularly the RF model, facilitated the understanding of spatial-temporal patterns of asthma and its association with air pollution parameters in Tehran. This knowledge can inform public health strategies aimed at reducing asthma prevalence and improving air quality. | (75) |
| 76 | 2021 | Iran | Physical disorders | Quantitative | Machine learning | the utilization of machine learning algorithms facilitated the identification of high-risk areas and correlated socio-economic land uses contributing to COVID-19 transmission in Tehran, Iran. These findings can inform targeted interventions and mitigation strategies to reduce the spread of the disease. | (76) |
| 77 | 2023 | Iran | Environmental issues | Quantitative | Machine learning | the study demonstrated the effectiveness of utilizing Artificial Intelligence, specifically ensemble machine learning models enhanced with evolutionary algorithms, for predicting dust susceptibility and creating dust susceptibility maps in the Bushehr province of Iran. | (77) |
| 78 | 2020 | Egypt | Physical disorders | Quantitative | Machine learning | the utilization of artificial intelligence in this context enhances the ability to understand, predict, and respond to the COVID-19 epidemic, thereby potentially reducing its impact on public health and society. | (78) |
| 79 | 2017 | Iran | Physical disorders | Quantitative | Machine learning | the utilization of AI in this survey enhances the ability to estimate survival rates for at-risk groups participating in colorectal cancer screening programs. It facilitates informed decision-making in healthcare planning and contributes to improving patient outcomes. | (79) |
| 80 | 2023 | Iran | Social disorders | Quantitative | Machine learning | the utilization of AI, particularly machine learning, in this study enables the analysis, classification, and prediction of Persian textual content related to DV against women in social media. It contributes to efforts aimed at addressing and mitigating the impact of DV by providing insights for intervention, support, and awareness-raising initiatives. | (80) |
| 81 | 2023 | Jordan | Physical disorders | Quantitative | Machine learning | the study provides valuable insights into the performance and limitations of AI-based conversational models like ChatGPT in diagnostic clinical microbiology scenarios. It emphasizes the importance of continued development and refinement to enhance the reliability and utility of AI in healthcare settings. | (81) |
| 82 | 2023 | Cyprus | Physical disorders | Quantitative | Machine learning | the study demonstrates the effectiveness of using AI, specifically the Random Forest model, in accurately detecting vitamin D status while addressing challenges such as multicollinearity in the dataset. These findings have implications for improving healthcare practices and reducing the burden associated with traditional biochemical tests. | (82) |
| 83 | 2015 | Iran | Physical disorders | Quantitative | Machine learning | the study demonstrates the effectiveness of utilizing AI, particularly the hybrid system incorporating AIRS and SVM, in improving the accuracy and speed of tuberculosis diagnosis. These outcomes have significant implications for enhancing healthcare practices and combating the global burden of tuberculosis. | (83) |
| 84 | 2016 | Iran | Physical disorders | Quantitative | Machine learning | the study highlights the effectiveness of utilizing AI, particularly the hybrid system incorporating real tournament selection mechanism into AIRS, in improving the accuracy of tuberculosis diagnosis. These outcomes have significant implications for enhancing healthcare practices and combating the global burden of tuberculosis. | (84) |
| 85 | 2024 | Egypt | Physical disorders | Quantitative | Machine learning | the outcomes of utilizing AI in this study contribute to the advancement of diagnostic methods for HCC by leveraging machine learning techniques to analyze circulating miRNAs. The proposed model demonstrates superior performance compared to traditional statistical approaches and identifies potential biomarkers for early diagnosis and prognosis of HCC. | (85) |
| 86 | 2023 | Iran | Physical disorders | Quantitative | Machine learning | the outcomes of utilizing AI in this study demonstrate the effectiveness of ML algorithms, particularly Random Forest, in predicting polypharmacy using health insurance claims data. These findings contribute to the development of predictive models for identifying individuals at risk of polypharmacy and guiding decision-making in healthcare. | (86) |
| 87 | 2022 | Iran | Physical disorders | Quantitative | Machine learning | The outcomes of utilizing Artificial Intelligence in this study contribute to the development of robust forecasting methodologies for COVID-19 new cases, aiding health authorities in Iran and potentially in other countries as well. The study highlights the effectiveness of hybrid models combining ANN with optimization algorithms, particularly ANN-FA, in accurately predicting COVID-19 trends. | (87) |
| 88 | 2023 | Iran | Physical disorders | Quantitative | Machine learning | the outcomes of utilizing Artificial Intelligence in this study highlight the potential of machine learning models in predicting mortality among COVID-19 patients with a history of smoking. These models can contribute to more effective management of patients and allocation of resources in healthcare settings. | (88) |
| 89 | 2021 | Iran | Physical disorders | Quantitative | Machine learning | the outcomes of utilizing Artificial Intelligence in this study demonstrate the effectiveness of machine learning techniques in predicting neonatal mortality in NICUs. These models have the potential to serve as valuable tools for healthcare providers in identifying high-risk neonates and implementing appropriate interventions to improve patient care. | (89) |
| 90 | 2019 | Iran | Physical disorders | Quantitative | Machine learning | the outcomes of utilizing Artificial Intelligence in this study demonstrate the effectiveness of data mining techniques, particularly the random forest model, in modeling and predicting brucellosis incidence data. These findings have implications for epidemiologists and public health authorities in their efforts to control and prevent brucellosis outbreaks. | (90) |
| 91 | 2021 | Iran | Environmental issues | Quantitative | Machine learning | the outcomes of utilizing Artificial Intelligence in this study provide valuable insights into the Spatio-temporal patterns of PM(2.5) pollution in Tehran and highlight the effectiveness of machine learning algorithms in modeling and predicting air pollution levels. These findings can inform urban planning and public health interventions aimed at mitigating the adverse effects of air pollution on the population. | (91) |
| 92 | 2024 | Iran | Physical disorders | Quantitative | Machine learning | the outcomes of utilizing Artificial Intelligence in this study provide valuable insights into predicting fasting blood glucose status using machine learning techniques. The developed model can aid in early diagnosis and treatment of diabetes, ultimately improving outcomes and quality of life for individuals at risk. | (92) |
| 93 | 2021 | UAE | Organizational issues | Quantitative | Machine learning | the utilization of Artificial Intelligence, particularly the random forests algorithm, facilitated the identification and understanding of key determinants of patient satisfaction, offering actionable insights for improving healthcare services. | (93) |
| 94 | 2022 | Iran | Organizational issues | Quantitative | Machine learning | the utilization of Artificial Intelligence, specifically LSTM-based deep learning models, offers promising results for improving the efficiency and effectiveness of palliative care service systems by accurately forecasting patient demand. | (94) |
| 95 | 2024 | Iran | Physical disorders | Quantitative | Machine learning | The study indicated that Neural Network (NN) and Random Forest (RF) were the best-performing ML-based approaches for predicting metastasis in CRC patients. The findings provide valuable insights into the predictive factors associated with metastasis in CRC patients, aiding in early detection and treatment planning. | (95) |
| 96 | 2023 | Iraq | Environmental issues | Quantitative | Machine learning | The study provided a methodology for forecasting the spatial variability of PM(2.5) concentration at high resolution during peak pollution months using freely available data. This methodology can be replicated in other regions for generating high-resolution PM(2.5) forecasting maps. | (96) |
| 97 | 2023 | Egypt | Physical disorders | Quantitative | Machine learning | the study highlights the impact of COVID-19 on patients' vital signs and infection severity, as well as the association between infection severity and physical work stress. However, the outcomes of utilizing AI are not mentioned in the text provided. | (97) |
| 98 | 2021 | Turkey | Physical disorders | Quantitative | Machine learning | the utilization of AI, specifically the auto-encoder based neural network model, demonstrates promising results for improving the detection of rare thyroid nodules in healthcare settings, leading to enhanced early detection of malign cases. | (98) |
| 99 | 2015 | Turkey | Physical disorders | Quantitative | Machine learning | the utilization of AI, particularly machine learning algorithms, in IVF treatment shows promise in improving the prediction of implantation outcomes for individual embryos, thereby enhancing the effectiveness of IVF procedures and optimizing patient care. | (99) |
| 100 | 2024 | Iran | Environmental issues | Quantitative | Machine learning | the study highlights the utility of AI, specifically machine learning methods, in predicting SOC levels and analyzing its distribution in regions impacted by mining. By leveraging various input variables and modeling techniques, AI can contribute to more accurate and efficient soil management practices, ultimately promoting soil health and sustainability. | (100) |

1. Abdesselam A, Zidoum H, Zadjali F, Hedjam R, Al-Ansari A, Bayoumi R, et al. Estimate of the HOMA-IR Cut-off Value for Identifying Subjects at Risk of Insulin Resistance Using a Machine Learning Approach. Sultan Qaboos Univ Med J. 2021;21(4):604-12.

2. Afrash MR, Bayani A, Shanbehzadeh M, Bahadori M, Kazemi-Arpanahi H. Developing the breast cancer risk prediction system using hybrid machine learning algorithms. J Educ Health Promot. 2022;11:272.

3. Akbulut A, Ertugrul E, Topcu V. Fetal health status prediction based on maternal clinical history using machine learning techniques. Comput Methods Programs Biomed. 2018;163:87-100.

4. Al Sadi K, Balachandran W. Revolutionizing Early Disease Detection: A High-Accuracy 4D CNN Model for Type 2 Diabetes Screening in Oman. Bioengineering (Basel). 2023;10(12).

5. Al Yammahi A, Aung Z. Forecasting the concentration of NO2 using statistical and machine learning methods: A case study in the UAE. Heliyon. 2023;9(2):e12584.

6. AlAgha AS, Faris H, Hammo BH, Al-Zoubi AM. Identifying β-thalassemia carriers using a data mining approach: The case of the Gaza Strip, Palestine. Artif Intell Med. 2018;88:70-83.

7. Albagmi FM, Hussain M, Kamal K, Sheikh MF, AlNujaidi HY, Bah S, et al. Predicting Multimorbidity Using Saudi Health Indicators (Sharik) Nationwide Data: Statistical and Machine Learning Approach. Healthcare (Basel). 2023;11(15).

8. AlKaabi LA, Ahmed LS, Al Attiyah MF, Abdel-Rahman ME. Predicting hypertension using machine learning: Findings from Qatar Biobank Study. PLoS One. 2020;15(10):e0240370.

9. Alrajhi AA, Alswailem OA, Wali G, Alnafee K, AlGhamdi S, Alarifi J, et al. Data-Driven Prediction for COVID-19 Severity in Hospitalized Patients. Int J Environ Res Public Health. 2022;19(5).

10. Alsayegh F, Alkhamis MA, Ali F, Attur S, Fountain-Jones NM, Zubaid M. Anemia or other comorbidities? using machine learning to reveal deeper insights into the drivers of acute coronary syndromes in hospital admitted patients. PLoS One. 2022;17(1):e0262997.

11. Alshalan R, Al-Khalifa H, Alsaeed D, Al-Baity H, Alshalan S. Detection of Hate Speech in COVID-19-Related Tweets in the Arab Region: Deep Learning and Topic Modeling Approach. J Med Internet Res. 2020;22(12):e22609.

12. AlShareedah A, Zidoum H, Al-Sawafi S, Al-Lawati B, Al-Ansari A. Machine Learning Approach for Predicting Systemic Lupus Erythematosus in an Oman-Based Cohort. Sultan Qaboos Univ Med J. 2023;23(3):328-35.

13. AlShehhi A, Almansoori TM, Alsuwaidi AR, Alblooshi H. Utilizing machine learning for survival analysis to identify risk factors for COVID-19 intensive care unit admission: A retrospective cohort study from the United Arab Emirates. PLoS One. 2024;19(1):e0291373.

14. AlShehhi A, Welsch R. Artificial intelligence for improving Nitrogen Dioxide forecasting of Abu Dhabi environment agency ground-based stations. J Big Data. 2023;10(1):92.

15. Amar LA, Taha AA, Mohamed MY. Prediction of the final size for COVID-19 epidemic using machine learning: A case study of Egypt. Infect Dis Model. 2020;5:622-34.

16. Amoori N, Cheraghian B, Amini P, Alavi SM. Identification of Risk Factors Associated with Tuberculosis in Southwest Iran: A Machine Learning Method. Med J Islam Repub Iran. 2024;38:5.

17. Ansaripour A, Zendehdel K, Tadayon N, Sadeghi F, Uyl-de Groot CA, Redekop WK. Use of data-mining to support real-world cost analyses: An example using HER2-positive breast cancer in Iran. PLoS One. 2018;13(10):e0205079.

18. Ayyoubzadeh SM, Ayyoubzadeh SM, Zahedi H, Ahmadi M, S RNK. Predicting COVID-19 Incidence Through Analysis of Google Trends Data in Iran: Data Mining and Deep Learning Pilot Study. JMIR Public Health Surveill. 2020;6(2):e18828.

19. Bagheri H, Tapak L, Karami M, Hosseinkhani Z, Najari H, Karimi S, Cheraghi Z. Forecasting the monthly incidence rate of brucellosis in west of Iran using time series and data mining from 2010 to 2019. PLoS One. 2020;15(5):e0232910.

20. Bashirian S, Mohammadi-Khoshnoud M, Khazaei S, Talebighane E, Keramat F, Bahreini F, et al. Identification of Risk Factors for COVID-19-related Death using Machine Learning Methods. Tanaffos. 2022;21(1):54-62.

21. Bodaghie M, Mahan F, Sahebi L, Dalili H. Neo-epidemiological machine learning based method for COVID-19 related estimations. PLoS One. 2023;18(3):e0263991.

22. Borhani F, Shafiepour Motlagh M, Rashidi Y, Ehsani AH. Estimation of short-lived climate forced sulfur dioxide in Tehran, Iran, using machine learning analysis. Stoch Environ Res Risk Assess. 2022;36(9):2847-60.

23. Choudhury A. Predicting cancer using supervised machine learning: Mesothelioma. Technol Health Care. 2021;29(1):45-58.

24. Dehdar S, Salimifard K, Mohammadi R, Marzban M, Saadatmand S, Fararouei M, Dianati-Nasab M. Applications of different machine learning approaches in prediction of breast cancer diagnosis delay. Front Oncol. 2023;13:1103369.

25. Demiray O, Gunes ED, Kulak E, Dogan E, Karaketir SG, Cifcili S, et al. Classification of patients with chronic disease by activation level using machine learning methods. Health Care Manag Sci. 2023;26(4):626-50.

26. Dianati-Nasab M, Salimifard K, Mohammadi R, Saadatmand S, Fararouei M, Hosseini KS, et al. Machine learning algorithms to uncover risk factors of breast cancer: insights from a large case-control study. Front Oncol. 2023;13:1276232.

27. Dikeç G, Oban V, Barış Usta M. Qualitative and Artificial Intelligence-Based Sentiment Analysis of Turkish Tweets Related to Schizophrenia. Turk Psikiyatri Derg. 2023;34(3):145-53.

28. Emiroglu M, Esin H, Erdogan M, Ugurlu L, Dursun A, Mertoglu S, et al. National study on use of artificial intelligence in breast disease and cancer. Bratisl Lek Listy. 2022;123(3):191-6.

29. Emre İ E, Erol N, Ayhan Y, Özkan Y, Erol Ç. The analysis of the effects of acute rheumatic fever in childhood on cardiac disease with data mining. Int J Med Inform. 2019;123:68-75.

30. Esmaeily H, Tayefi M, Ghayour-Mobarhan M, Amirabadizadeh A. Comparing Three Data Mining Algorithms for Identifying the Associated Risk Factors of Type 2 Diabetes. Iran Biomed J. 2018;22(5):303-11.

31. F A, C S, L A. Supervised Algorithms of Machine Learning for the Prediction of Cervical Cancer. J Biomed Phys Eng. 2020;10(4):513-22.

32. Farran B, AlWotayan R, Alkandari H, Al-Abdulrazzaq D, Channanath A, Thanaraj TA. Use of Non-invasive Parameters and Machine-Learning Algorithms for Predicting Future Risk of Type 2 Diabetes: A Retrospective Cohort Study of Health Data From Kuwait. Front Endocrinol (Lausanne). 2019;10:624.

33. Farran B, Channanath AM, Behbehani K, Thanaraj TA. Predictive models to assess risk of type 2 diabetes, hypertension and comorbidity: machine-learning algorithms and validation using national health data from Kuwait--a cohort study. BMJ Open. 2013;3(5).

34. ForouzeshFar P, Safaei AA, Ghaderi F, Hashemikamangar SS. Dental Caries diagnosis from bitewing images using convolutional neural networks. BMC Oral Health. 2024;24(1):211.

35. Fryan LHA, Alazzam MB. Survival Analysis of Oncological Patients Using Machine Learning Method. Healthcare (Basel). 2022;11(1).

36. Ghany KKA, Zawbaa HM, Sabri HM. COVID-19 prediction using LSTM algorithm: GCC case study. Inform Med Unlocked. 2021;23:100566.

37. Gholami H, Mohammadifar A, Behrooz RD, Kaskaoutis DG, Li Y, Song Y. Intrinsic and extrinsic techniques for quantification uncertainty of an interpretable GRU deep learning model used to predict atmospheric total suspended particulates (TSP) in Zabol, Iran during the dusty period of 120-days wind. Environ Pollut. 2024;342:123082.

38. Giannakopoulos K, Kavadella A, Aaqel Salim A, Stamatopoulos V, Kaklamanos EG. Evaluation of the Performance of Generative AI Large Language Models ChatGPT, Google Bard, and Microsoft Bing Chat in Supporting Evidence-Based Dentistry: Comparative Mixed Methods Study. J Med Internet Res. 2023;25:e51580.

39. Göksel P, Oban V, Dikeç G, Usta MB. Qualitative and Artificial Intelligence-Based Sentiment Analysis of Turkish Twitter Messages Related to Autism Spectrum Disorders. Cureus. 2023;15(5):e38446.

40. Günal E, Budak M, Kılıç M, Cemek B, Sırrı M. Combining spatial autocorrelation with artificial intelligence models to estimate spatial distribution and risks of heavy metal pollution in agricultural soils. Environ Monit Assess. 2023;195(2):317.

41. Habib M, Faris M, Qaddoura R, Alomari M, Alomari A, Faris H. Toward an Automatic Quality Assessment of Voice-Based Telemedicine Consultations: A Deep Learning Approach. Sensors (Basel). 2021;21(9).

42. Habibzadeh A, Khademolhosseini S, Kouhpayeh A, Niakan A, Asadi MA, Ghasemi H, et al. Machine learning-based models to predict the need for neurosurgical intervention after moderate traumatic brain injury. Health Sci Rep. 2023;6(11):e1666.

43. Hameed Abdulkareem K, Awad Mutlag A, Musa Dinar A, Frnda J, Abed Mohammed M, Hasan Zayr F, et al. Smart Healthcare System for Severity Prediction and Critical Tasks Management of COVID-19 Patients in IoT-Fog Computing Environments. Comput Intell Neurosci. 2022;2022:5012962.

44. Hammoudi Halat D, Abdel-Salam AG, Bensaid A, Soltani A, Alsarraj L, Dalli R, Malki A. Use of machine learning to assess factors affecting progression, retention, and graduation in first-year health professions students in Qatar: a longitudinal study. BMC Med Educ. 2023;23(1):909.

45. Hassanzadeh R, Farhadian M, Rafieemehr H. Hospital mortality prediction in traumatic injuries patients: comparing different SMOTE-based machine learning algorithms. BMC Med Res Methodol. 2023;23(1):101.

46. Hosseini Sarkhosh SM, Esteghamati A, Hemmatabadi M, Daraei M. Predicting diabetic nephropathy in type 2 diabetic patients using machine learning algorithms. J Diabetes Metab Disord. 2022;21(2):1433-41.

47. Huang W, Ao S, Han D, Liu Y, Liu S, Huang Y. Data-Driven and Machine-Learning Methods to Project Coronavirus Disease 2019 Pandemic Trend in Eastern Mediterranean. Front Public Health. 2021;9:602353.

48. Jamei M, Ahmadianfar I, Karbasi M, Jawad AH, Farooque AA, Yaseen ZM. The assessment of emerging data-intelligence technologies for modeling Mg(+2) and SO(4)(-2) surface water quality. J Environ Manage. 2021;300:113774.

49. John M, Shaiba H. Main factors influencing recovery in MERS Co-V patients using machine learning. J Infect Public Health. 2019;12(5):700-4.

50. Kashef A, Khatibi T, Mehrvar A. Prediction of Cranial Radiotherapy Treatment in Pediatric Acute Lymphoblastic Leukemia Patients Using Machine Learning: A Case Study at MAHAK Hospital. Asian Pac J Cancer Prev. 2020;21(11):3211-9.

51. Keser G, Bayrakdar İ, Pekiner FN, Çelik Ö, Orhan K. A deep learning algorithm for classification of oral lichen planus lesions from photographic images: A retrospective study. J Stomatol Oral Maxillofac Surg. 2023;124(1):101264.

52. Khan W, Zaki N, Masud MM, Ahmad A, Ali L, Ali N, Ahmed LA. Infant birth weight estimation and low birth weight classification in United Arab Emirates using machine learning algorithms. Sci Rep. 2022;12(1):12110.

53. Khounraz F, Khodadoost M, Gholamzadeh S, Pourhamidi R, Baniasadi T, Jafarbigloo A, et al. Prognosis of COVID-19 patients using lab tests: A data mining approach. Health Sci Rep. 2023;6(1):e1049.

54. Koç E, Türkoğlu M. Forecasting of medical equipment demand and outbreak spreading based on deep long short-term memory network: the COVID-19 pandemic in Turkey. Signal Image Video Process. 2022;16(3):613-21.

55. Li J, Garshick E, Hart JE, Li L, Shi L, Al-Hemoud A, et al. Estimation of ambient PM(2.5) in Iraq and Kuwait from 2001 to 2018 using machine learning and remote sensing. Environ Int. 2021;151:106445.

56. Li J, Kang CM, Wolfson JM, Alahmad B, Al-Hemoud A, Garshick E, Koutrakis P. Estimation of fine particulate matter in an arid area from visibility based on machine learning. J Expo Sci Environ Epidemiol. 2022;32(6):926-31.

57. Mahboobi H, Shakiba A, Mirbagheri B. Improving groundwater nitrate concentration prediction using local ensemble of machine learning models. J Environ Manage. 2023;345:118782.

58. Marzouk M, Elshaboury N, Abdel-Latif A, Azab S. Deep learning model for forecasting COVID-19 outbreak in Egypt. Process Saf Environ Prot. 2021;153:363-75.

59. Mehrnoush V, Ranjbar A, Farashah MV, Darsareh F, Shekari M, Jahromi MS. Prediction of postpartum hemorrhage using traditional statistical analysis and a machine learning approach. AJOG Glob Rep. 2023;3(2):100185.

60. Mohammadi G, Azizmohammad Looha M, Pourhoseingholi MA, Rezaei Tavirani M, Sohrabi S, Zareie Shab Khaneh A, et al. Classification and Diagnostic Prediction of Colorectal Cancer Mortality Based on Machine Learning Algorithms: A Multicenter National Study. Asian Pac J Cancer Prev. 2024;25(1):333-42.

61. Mohammadnezhad K, Sahebi MR, Alatab S, Sadjadi A. Modeling Epidemiology Data with Machine Learning Technique to Detect Risk Factors for Gastric Cancer. J Gastrointest Cancer. 2023.

62. Mohammadpour A, Keshtkar M, Samaei MR, Isazadeh S, Mousavi Khaneghah A. Assessing water quality index and health risk using deterministic and probabilistic approaches in Darab County, Iran; A machine learning for fluoride prediction. Chemosphere. 2024;352:141284.

63. Momahhed SS, Sefiddashti SE, Minaei B, Arab M. The optimal co-insurance rate for outpatient drug expenses of Iranian health insured based on the data mining method. Int J Equity Health. 2024;23(1):25.

64. Mosayebi A, Mojaradi B, Bonyadi Naeini A, Khodadad Hosseini SH. Modeling and comparing data mining algorithms for prediction of recurrence of breast cancer. PLoS One. 2020;15(10):e0237658.

65. Moslehi S, Rabiei N, Soltanian AR, Mamani M. Application of machine learning models based on decision trees in classifying the factors affecting mortality of COVID-19 patients in Hamadan, Iran. BMC Med Inform Decis Mak. 2022;22(1):192.

66. Mousa KM, Mousa FA, Mohamed HS, Elsawy MM. Prediction of Foot Ulcers Using Artificial Intelligence for Diabetic Patients at Cairo University Hospital, Egypt. SAGE Open Nurs. 2023;9:23779608231185873.

67. Naghavi A, Teismann T, Asgari Z, Mohebbian MR, Mansourian M, Mañanas M. Accurate Diagnosis of Suicide Ideation/Behavior Using Robust Ensemble Machine Learning: A University Student Population in the Middle East and North Africa (MENA) Region. Diagnostics (Basel). 2020;10(11).

68. Nopour R. Prediction of five-year survival among esophageal cancer patients using machine learning. Heliyon. 2023;9(12):e22654.

69. Nourelahi M, Dadboud F, Khalili H, Niakan A, Parsaei H. A machine learning model for predicting favorable outcome in severe traumatic brain injury patients after 6 months. Acute Crit Care. 2022;37(1):45-52.

70. Qasrawi R, Amro M, VicunaPolo S, Abu Al-Halawa D, Agha H, Abu Seir R, et al. Machine learning techniques for predicting depression and anxiety in pregnant and postpartum women during the COVID-19 pandemic: a cross-sectional regional study. F1000Res. 2022;11:390.

71. Qasrawi R, Hoteit M, Tayyem R, Bookari K, Al Sabbah H, Kamel I, et al. Machine learning techniques for the identification of risk factors associated with food insecurity among adults in Arab countries during the COVID-19 pandemic. BMC Public Health. 2023;23(1):1805.

72. Qasrawi R, Vicuna Polo S, Abu Khader R, Abu Al-Halawa D, Hallaq S, Abu Halaweh N, Abdeen Z. Machine learning techniques for identifying mental health risk factor associated with schoolchildren cognitive ability living in politically violent environments. Front Psychiatry. 2023;14:1071622.

73. Ranjbar A, Montazeri F, Farashah MV, Mehrnoush V, Darsareh F, Roozbeh N. Machine learning-based approach for predicting low birth weight. BMC Pregnancy Childbirth. 2023;23(1):803.

74. Rashed AM, El-Attar NE, Abdelminaam DS, Abdelfatah M. Analysis the patients' careflows using process mining. PLoS One. 2023;18(2):e0281836.

75. Razavi-Termeh SV, Sadeghi-Niaraki A, Choi SM. Effects of air pollution in Spatio-temporal modeling of asthma-prone areas using a machine learning model. Environ Res. 2021;200:111344.

76. Razavi-Termeh SV, Sadeghi-Niaraki A, Farhangi F, Choi SM. COVID-19 Risk Mapping with Considering Socio-Economic Criteria Using Machine Learning Algorithms. Int J Environ Res Public Health. 2021;18(18).

77. Razavi-Termeh SV, Sadeghi-Niaraki A, Naqvi RA, Choi SM. Dust detection and susceptibility mapping by aiding satellite imagery time series and integration of ensemble machine learning with evolutionary algorithms. Environ Pollut. 2023;335:122241.

78. Saba AI, Elsheikh AH. Forecasting the prevalence of COVID-19 outbreak in Egypt using nonlinear autoregressive artificial neural networks. Process Saf Environ Prot. 2020;141:1-8.

79. Safdari R, Maserat E, Asadzadeh Aghdaei H, Javan Amoli AH, Mohaghegh Shalmani H. Person centered prediction of survival in population based screening program by an intelligent clinical decision support system. Gastroenterol Hepatol Bed Bench. 2017;10(1):60-5.

80. Salehi M, Ghahari S, Hosseinzadeh M, Ghalichi L. Domestic violence risk prediction in Iran using a machine learning approach by analyzing Persian textual content in social media. Heliyon. 2023;9(5):e15667.

81. Sallam M, Al-Salahat K, Al-Ajlouni E. ChatGPT Performance in Diagnostic Clinical Microbiology Laboratory-Oriented Case Scenarios. Cureus. 2023;15(12):e50629.

82. Sancar N, Tabrizi SS. Machine learning approach for the detection of vitamin D level: a comparative study. BMC Med Inform Decis Mak. 2023;23(1):219.

83. Saybani MR, Shamshirband S, Golzari Hormozi S, Wah TY, Aghabozorgi S, Pourhoseingholi MA, Olariu T. Diagnosing tuberculosis with a novel support vector machine-based artificial immune recognition system. Iran Red Crescent Med J. 2015;17(4):e24557.

84. Saybani MR, Shamshirband S, Golzari S, Wah TY, Saeed A, Mat Kiah ML, Balas VE. RAIRS2 a new expert system for diagnosing tuberculosis with real-world tournament selection mechanism inside artificial immune recognition system. Med Biol Eng Comput. 2016;54(2-3):385-99.

85. Sayed GI, Solyman M, El Gedawy G, Moemen YS, Aboul-Ella H, Hassanien AE. Circulating miRNA's biomarkers for early detection of hepatocellular carcinoma in Egyptian patients based on machine learning algorithms. Sci Rep. 2024;14(1):4989.

86. Seyedtabib M, Kamyari N. Predicting polypharmacy in half a million adults in the Iranian population: comparison of machine learning algorithms. BMC Med Inform Decis Mak. 2023;23(1):84.

87. Shaibani MJ, Emamgholipour S, Moazeni SS. Investigation of robustness of hybrid artificial neural network with artificial bee colony and firefly algorithm in predicting COVID-19 new cases: case study of Iran. Stoch Environ Res Risk Assess. 2022;36(9):2461-76.

88. Sharifi-Kia A, Nahvijou A, Sheikhtaheri A. Machine learning-based mortality prediction models for smoker COVID-19 patients. BMC Med Inform Decis Mak. 2023;23(1):129.

89. Sheikhtaheri A, Zarkesh MR, Moradi R, Kermani F. Prediction of neonatal deaths in NICUs: development and validation of machine learning models. BMC Med Inform Decis Mak. 2021;21(1):131.

90. Shirmohammadi-Khorram N, Tapak L, Hamidi O, Maryanaji Z. A comparison of three data mining time series models in prediction of monthly brucellosis surveillance data. Zoonoses Public Health. 2019;66(7):759-72.

91. Shogrkhodaei SZ, Razavi-Termeh SV, Fathnia A. Spatio-temporal modeling of PM(2.5) risk mapping using three machine learning algorithms. Environ Pollut. 2021;289:117859.

92. Shojaee-Mend H, Velayati F, Tayefi B, Babaee E. Prediction of Diabetes Using Data Mining and Machine Learning Algorithms: A Cross-Sectional Study. Healthc Inform Res. 2024;30(1):73-82.

93. Simsekler MCE, Alhashmi NH, Azar E, King N, Luqman R, Al Mulla A. Exploring drivers of patient satisfaction using a random forest algorithm. BMC Med Inform Decis Mak. 2021;21(1):157.

94. Soltani M, Farahmand M, Pourghaderi AR. Machine learning-based demand forecasting in cancer palliative care home hospitalization. J Biomed Inform. 2022;130:104075.

95. Talebi R, Celis-Morales CA, Akbari A, Talebi A, Borumandnia N, Pourhoseingholi MA. Machine learning-based classifiers to predict metastasis in colorectal cancer patients. Front Artif Intell. 2024;7:1285037.

96. Tao H, Jawad AH, Shather AH, Al-Khafaji Z, Rashid TA, Ali M, et al. Machine learning algorithms for high-resolution prediction of spatiotemporal distribution of air pollution from meteorological and soil parameters. Environ Int. 2023;175:107931.

97. Torad AA, Shamy FE, Kadry AM, Ahmed ZS. Using Machine Learning Models To Investigate The Relationship Between Corporeal Workload And Clinical And Epidemiological Features Of Patients Infected With COVID-19 In Egypt. J Pak Med Assoc. 2023;73(Suppl 4)(4):S242-s6.

98. Turk G, Ozdemir M, Zeydan R, Turk Y, Bilgin Z, Zeydan E. On the identification of thyroid nodules using semi-supervised deep learning. Int J Numer Method Biomed Eng. 2021;37(3):e3433.

99. Uyar A, Bener A, Ciray HN. Predictive Modeling of Implantation Outcome in an In Vitro Fertilization Setting: An Application of Machine Learning Methods. Med Decis Making. 2015;35(6):714-25.

100. Zhang T, Li Y, Wang M. Remote sensing-based prediction of organic carbon in agricultural and natural soils influenced by salt and sand mining using machine learning. J Environ Manage. 2024;352:120107.
