# Supplementary material for: A Systematic Review of the Outcomes of Utilization of Artificial Intelligence Within the Healthcare Systems of the Middle East: A Thematic Analysis of Findings
Source: Health Sci Rep. 2024 Dec 24;7(12):e70300. doi: 10.1002/hsr2.70300 (PMC11667773; doi:10.1002/hsr2.70300)
Supplement: Supplementary file 2 — Supporting information. [file HSR2-7-e70300-s002.docx]

| **Appendix 2 .** The result of quality assessment of selected articles based on the AACODS^[[1]](#footnote-1)^ checklist | | | | | | | | |
| --- | --- | --- | --- | --- | --- | --- | --- | --- |
| **Number** | **Author(s)/ Citation** | **Q1** | **Q2** | **Q3** | **Q4** | **Q5** | **Q6** | **Score** |
|  | Abdesselam/(1) | 2 | 0 | 2 | 2 | 2 | 2 | 10 |
|  | Afrash/(2) | 2 | 2 | 2 | 2 | 2 | 2 | 12 |
|  | Akbulut/(3) | 2 | 2 | 2 | 2 | 1 | 2 | 11 |
|  | Sadi/(4) | 2 | 2 | 2 | 2 | 2 | 2 | 12 |
|  | Yammahi/(5) | 2 | 2 | 2 | 2 | 2 | 2 | 12 |
|  | AlAgha/(6) | 2 | 2 | 2 | 2 | 1 | 2 | 11 |
|  | Albagmi/(7) | 2 | 2 | 2 | 2 | 2 | 2 | 12 |
|  | AlKaabi/(8) | 2 | 2 | 2 | 2 | 2 | 2 | 12 |
|  | Alrajhi/(9) | 2 | 2 | 2 | 2 | 2 | 2 | 12 |
|  | Alsayegh/(10) | 2 | 0 | 2 | 2 | 2 | 2 | 10 |
|  | Alshalan/(11) | 2 | 2 | 2 | 2 | 2 | 2 | 12 |
|  | AlShareedah/(12) | 2 | 2 | 1 | 1 | 2 | 2 | 10 |
|  | AlShehhi/(13) | 2 | 2 | 2 | 2 | 2 | 2 | 12 |
|  | AlShehhi/(14) | 2 | 2 | 2 | 2 | 2 | 2 | 12 |
|  | Amar/(15) | 2 | 2 | 2 | 1 | 2 | 1 | 11 |
|  | Amoori/(16) | 2 | 2 | 2 | 2 | 2 | 2 | 12 |
|  | Ansaripour/(17) | 2 | 2 | 2 | 2 | 1 | 2 | 11 |
|  | Ayyoubzadeh/(18) | 2 | 1 | 1 | 2 | 2 | 2 | 10 |
|  | Bagheri/(19) | 2 | 2 | 2 | 2 | 2 | 2 | 12 |
|  | Bashirian/(20) | 2 | 1 | 2 | 2 | 2 | 2 | 11 |
|  | Bodaghie/(21) | 2 | 2 | 2 | 2 | 2 | 1 | 11 |
|  | Borhani/(22) | 2 | 2 | 2 | 1 | 1 | 2 | 10 |
|  | Choudhury/(23) | 2 | 2 | 2 | 1 | 1 | 2 | 10 |
|  | Dehdar/(24) | 2 | 2 | 2 | 2 | 2 | 2 | 12 |
|  | Demiray/(25) | 2 | 2 | 2 | 2 | 2 | 2 | 12 |
|  | Dianati-Nasab/(26) | 2 | 2 | 2 | 1 | 2 | 2 | 11 |
|  | Dikeç/(27) | 2 | 2 | 2 | 2 | 2 | 2 | 12 |
|  | Emiroglu/(28) | 1 | 2 | 2 | 2 | 0 | 2 | 9 |
|  | Emre/(29) | 2 | 2 | 2 | 2 | 1 | 2 | 11 |
|  | Esmaeily/(30) | 2 | 2 | 2 | 2 | 1 | 2 | 11 |
|  | Asadi/(31) | 2 | 2 | 2 | 2 | 0 | 2 | 10 |
|  | Farran/(32) | 2 | 2 | 2 | 2 | 2 | 2 | 12 |
|  | Farran/(33) | 2 | 1 | 2 | 2 | 0 | 2 | 9 |
|  | ForouzeshFar/(34) | 2 | 2 | 2 | 2 | 2 | 2 | 12 |
|  | Fryan/(35) | 2 | 2 | 2 | 2 | 2 | 2 | 12 |
|  | Ghany/(36) | 2 | 2 | 2 | 2 | 2 | 2 | 12 |
|  | Gholami/(37) | 2 | 2 | 2 | 2 | 2 | 2 | 12 |
|  | Giannakopoulos/(38) | 2 | 2 | 2 | 2 | 2 | 2 | 12 |
|  | Göksel/(39) | 2 | 2 | 2 | 2 | 2 | 2 | 12 |
|  | Günal/(40) | 2 | 2 | 2 | 2 | 2 | 2 | 12 |
|  | Habib/(41) | 2 | 2 | 2 | 2 | 2 | 2 | 12 |
|  | Habibzadeh/(42) | 2 | 2 | 2 | 2 | 2 | 2 | 12 |
|  | Abdulkareem/(43) | 2 | 1 | 1 | 1 | 1 | 1 | 7 |
|  | Hammoudi/(44) | 2 | 2 | 2 | 2 | 2 | 2 | 12 |
|  | Hassanzadeh/(45) | 2 | 2 | 2 | 2 | 2 | 2 | 12 |
|  | Sarkhosh/(46) | 2 | 2 | 2 | 2 | 2 | 2 | 12 |
|  | Huang/(47) | 2 | 2 | 2 | 2 | 1 | 2 | 11 |
|  | Jamei/(48) | 2 | 2 | 2 | 2 | 2 | 2 | 12 |
|  | John/(49) | 2 | 2 | 2 | 2 | 1 | 2 | 11 |
|  | Kashef/(50) | 2 | 2 | 2 | 2 | 1 | 2 | 11 |
|  | Keser/(51) | 2 | 2 | 2 | 2 | 2 | 2 | 12 |
|  | Khan/(52) | 2 | 2 | 2 | 2 | 2 | 2 | 12 |
|  | Khounraz/(53) | 2 | 2 | 2 | 2 | 2 | 2 | 12 |
|  | Koç/(54) | 2 | 2 | 2 | 2 | 2 | 2 | 12 |
|  | Li-1/(55) | 2 | 2 | 2 | 2 | 1 | 2 | 11 |
|  | Li-2/(56) | 2 | 2 | 2 | 2 | 2 | 2 | 12 |
|  | Mahboobi/(57) | 2 | 2 | 2 | 2 | 2 | 2 | 12 |
|  | Marzouk/(58) | 2 | 2 | 2 | 2 | 2 | 2 | 12 |
|  | Mehrnoush/(59) | 2 | 2 | 2 | 2 | 2 | 2 | 12 |
|  | Mohammadi/(60) | 2 | 2 | 2 | 2 | 2 | 2 | 12 |
|  | Mohammadnezhad/(61) | 2 | 2 | 2 | 2 | 2 | 2 | 12 |
|  | Mohammadpour/(62) | 2 | 2 | 2 | 2 | 2 | 2 | 12 |
|  | Momahhed/(63) | 2 | 2 | 2 | 2 | 2 | 2 | 12 |
|  | Mosayebi/(64) | 2 | 2 | 2 | 2 | 1 | 2 | 11 |
|  | Moslehi/(65) | 2 | 2 | 2 | 2 | 2 | 2 | 12 |
|  | Mousa/(66) | 2 | 2 | 2 | 2 | 2 | 2 | 12 |
|  | Naghavi/(67) | 2 | 2 | 2 | 2 | 2 | 2 | 12 |
|  | Nopour/(68) | 2 | 2 | 2 | 2 | 2 | 2 | 12 |
|  | Nourelahi/(69) | 2 | 2 | 2 | 2 | 2 | 2 | 12 |
|  | Qasrawi1/(70) | 2 | 2 | 2 | 2 | 2 | 2 | 12 |
|  | Qasrawi2/(71) | 2 | 2 | 2 | 2 | 2 | 2 | 12 |
|  | Qasrawi3/(72) | 2 | 2 | 2 | 2 | 2 | 2 | 12 |
|  | Ranjbar/(73) | 2 | 2 | 2 | 2 | 2 | 2 | 12 |
|  | Rashed/(74) | 2 | 1 | 2 | 2 | 2 | 2 | 11 |
|  | Razavi1/(75) | 2 | 2 | 2 | 2 | 2 | 2 | 12 |
|  | Razavi2/(76) | 2 | 2 | 2 | 2 | 2 | 2 | 12 |
|  | Razavi3/(77) | 2 | 2 | 2 | 2 | 2 | 2 | 12 |
|  | Saba/(78) | 2 | 2 | 2 | 2 | 2 | 2 | 12 |
|  | Safdari/(79) | 2 | 1 | 2 | 2 | 1 | 2 | 10 |
|  | Salehi/(80) | 2 | 2 | 2 | 2 | 2 | 2 | 12 |
|  | Sallam/(81) | 2 | 2 | 2 | 2 | 2 | 2 | 12 |
|  | Sancar/(82) | 2 | 2 | 2 | 2 | 2 | 2 | 12 |
|  | Saybani/(83) | 2 | 1 | 2 | 1 | 0 | 2 | 8 |
|  | Saybani/(84) | 2 | 2 | 2 | 1 | 0 | 2 | 9 |
|  | Sayed/(85) | 2 | 2 | 2 | 2 | 2 | 2 | 12 |
|  | Seyedtabib/(86) | 2 | 1 | 2 | 2 | 2 | 2 | 11 |
|  | Shaibani/(87) | 2 | 2 | 1 | 2 | 2 | 1 | 10 |
|  | Sharifi/(88) | 2 | 2 | 2 | 2 | 2 | 2 | 12 |
|  | Sheikhtaheri/(89) | 2 | 2 | 2 | 2 | 1 | 2 | 11 |
|  | Shirmohammadi/(90) | 2 | 2 | 2 | 2 | 1 | 2 | 11 |
|  | Shogrkhodaei/(91) | 2 | 2 | 2 | 2 | 1 | 2 | 11 |
|  | Shojaee/(92) | 2 | 2 | 2 | 2 | 2 | 2 | 12 |
|  | Simsekler/(93) | 2 | 1 | 2 | 2 | 1 | 2 | 10 |
|  | Soltani/(94) | 2 | 2 | 2 | 2 | 2 | 2 | 12 |
|  | Talebi/(95) | 2 | 2 | 2 | 2 | 2 | 2 | 12 |
|  | Tao/(96) | 2 | 2 | 2 | 2 | 2 | 2 | 12 |
|  | Torad/(97) | 2 | 2 | 2 | 2 | 2 | 2 | 12 |
|  | Turk/(98) | 2 | 2 | 2 | 2 | 2 | 2 | 12 |
|  | Uyar/(99) | 2 | 2 | 2 | 2 | 0 | 2 | 10 |
|  | Zhang/(100) | 2 | 2 | 2 | 2 | 2 | 2 | 12 |
| Scoring AACODS checklist questions: Yes =2, Can’t Tell= 1, No = 0 | | | | | | | | |
| Q1: Authority: Is the author or source of the information reputable and trustworthy?  Q2: Accuracy: Is the information reliable, truthful, and correct?  Q3: Coverage: Does the information cover the topic comprehensively and sufficiently?  Q4: Objectivity: Is the information presented in an unbiased and balanced way?  Q5: Date: Is the information current and up-to-date?  Q6: Significance: Is the information relevant, important, and valuable to the topic? | | | | | | | | |

1. Abdesselam A, Zidoum H, Zadjali F, Hedjam R, Al-Ansari A, Bayoumi R, et al. Estimate of the HOMA-IR cut-off value for identifying subjects at risk of insulin resistance using a machine learning approach. Sultan Qaboos University Medical Journal. 2021;21(4):604.

2. Afrash MR, Bayani A, Shanbehzadeh M, Bahadori M, Kazemi-Arpanahi H. Developing the breast cancer risk prediction system using hybrid machine learning algorithms. Journal of Education and Health Promotion. 2022;11(1):272.

3. Akbulut A, Ertugrul E, Topcu V. Fetal health status prediction based on maternal clinical history using machine learning techniques. Computer methods and programs in biomedicine. 2018;163:87-100.

4. Al Sadi K, Balachandran W. Revolutionizing Early Disease Detection: A High-Accuracy 4D CNN Model for Type 2 Diabetes Screening in Oman. Bioengineering. 2023;10(12):1420.

5. Al Yammahi A, Aung Z. Forecasting the concentration of NO2 using statistical and machine learning methods: A case study in the UAE. Heliyon. 2023;9(2).

6. AlAgha AS, Faris H, Hammo BH, Ala’M A-Z. Identifying β-thalassemia carriers using a data mining approach: The case of the Gaza Strip, Palestine. Artificial intelligence in medicine. 2018;88:70-83.

7. Albagmi FM, Hussain M, Kamal K, Sheikh MF, AlNujaidi HY, Bah S, et al., editors. Predicting Multimorbidity Using Saudi Health Indicators (Sharik) Nationwide Data: Statistical and Machine Learning Approach. Healthcare; 2023: MDPI.

8. AlKaabi LA, Ahmed LS, Al Attiyah MF, Abdel-Rahman ME. Predicting hypertension using machine learning: Findings from Qatar Biobank Study. Plos one. 2020;15(10):e0240370.

9. Alrajhi AA, Alswailem OA, Wali G, Alnafee K, AlGhamdi S, Alarifi J, et al. Data-driven prediction for COVID-19 severity in hospitalized patients. International journal of environmental research and public health. 2022;19(5):2958.

10. Alsayegh F, Alkhamis MA, Ali F, Attur S, Fountain-Jones NM, Zubaid M. Anemia or other comorbidities? using machine learning to reveal deeper insights into the drivers of acute coronary syndromes in hospital admitted patients. Plos one. 2022;17(1):e0262997.

11. Alshalan R, Al-Khalifa H, Alsaeed D, Al-Baity H, Alshalan S. Detection of hate speech in covid-19–related tweets in the arab region: Deep learning and topic modeling approach. Journal of Medical Internet Research. 2020;22(12):e22609.

12. AlShareedah A, Zidoum H, Al-Sawafi S, Al-Lawati B, Al-Ansari A. Machine Learning Approach for Predicting Systemic Lupus Erythematosus in an Oman-Based Cohort. Sultan Qaboos University Medical Journal. 2023;23(3):328.

13. AlShehhi A, Almansoori TM, Alsuwaidi AR, Alblooshi H. Utilizing machine learning for survival analysis to identify risk factors for COVID-19 intensive care unit admission: A retrospective cohort study from the United Arab Emirates. Plos one. 2024;19(1):e0291373.

14. AlShehhi A, Welsch R. Artificial intelligence for improving Nitrogen Dioxide forecasting of Abu Dhabi environment agency ground-based stations. Journal of Big Data. 2023;10(1):92.

15. Amar LA, Taha AA, Mohamed MY. Prediction of the final size for COVID-19 epidemic using machine learning: A case study of Egypt. Infectious Disease Modelling. 2020;5:622-34.

16. Amoori N, Cheraghian B, Amini P, Alavi SM. Identification of Risk Factors Associated with Tuberculosis in Southwest Iran: A Machine Learning Method. Medical Journal of the Islamic Republic of Iran. 2024;38.

17. Ansaripour A, Zendehdel K, Tadayon N, Sadeghi F, Uyl-de Groot CA, Redekop WK. Use of data-mining to support real-world cost analyses: An example using HER2-positive breast cancer in Iran. PLoS One. 2018;13(10):e0205079.

18. Ayyoubzadeh SM, Ayyoubzadeh SM, Zahedi H, Ahmadi M, Kalhori SRN. Predicting COVID-19 incidence through analysis of google trends data in Iran: data mining and deep learning pilot study. JMIR public health and surveillance. 2020;6(2):e18828.

19. Bagheri H, Tapak L, Karami M, Hosseinkhani Z, Najari H, Karimi S, Cheraghi Z. Forecasting the monthly incidence rate of brucellosis in west of Iran using time series and data mining from 2010 to 2019. PloS one. 2020;15(5):e0232910.

20. Bashirian S, Mohammadi-Khoshnoud M, Khazaei S, Talebighane E, Keramat F, Bahreini F, et al. Identification of Risk Factors for COVID-19-related Death using Machine Learning Methods. Tanaffos. 2022;21(1):54.

21. Bodaghie M, Mahan F, Sahebi L, Dalili H. Neo-epidemiological machine learning based method for COVID-19 related estimations. Plos one. 2023;18(3):e0263991.

22. Borhani F, Shafiepour Motlagh M, Rashidi Y, Ehsani AH. Estimation of short-lived climate forced sulfur dioxide in Tehran, Iran, using machine learning analysis. Stochastic Environmental Research and Risk Assessment. 2022:1-14.

23. Choudhury A. Predicting cancer using supervised machine learning: Mesothelioma. Technology and Health Care. 2021;29(1):45-58.

24. Dehdar S, Salimifard K, Mohammadi R, Marzban M, Saadatmand S, Fararouei M, Dianati-Nasab M. Applications of different machine learning approaches in prediction of breast cancer diagnosis delay. Frontiers in Oncology. 2023;13:1103369.

25. Demiray O, Gunes ED, Kulak E, Dogan E, Karaketir SG, Cifcili S, et al. Classification of patients with chronic disease by activation level using machine learning methods. Health Care Management Science. 2023;26(4):626-50.

26. Dianati-Nasab M, Salimifard K, Mohammadi R, Saadatmand S, Fararouei M, Hosseini KS, et al. Machine learning algorithms to uncover risk factors of breast cancer: insights from a large case-control study. Frontiers in Oncology. 2023;13.

27. Dikeç G, Oban V, Usta MB. Qualitative and Artificial Intelligence-Based Sentiment Analysis of Turkish Tweets Related to Schizophrenia. Turkish Journal of Psychiatry. 2023;34(3):145.

28. Emiroglu M, Esin H, Erdogan M, Ugurlu L, Dursun A, Mertoglu S, et al. National study on use of artificial intelligence in breast disease and cancer. ARTIFICIAL INTELLIGENCE. 2022;191:196.

29. Emre IE, Erol N, Ayhan YI, Özkan Y, Erol Ç. The analysis of the effects of acute rheumatic fever in childhood on cardiac disease with data mining. International journal of medical informatics. 2019;123:68-75.

30. Esmaeily H, Tayefi M, Ghayour-Mobarhan M, Amirabadizadeh A. Comparing three data mining algorithms for identifying the associated risk factors of type 2 diabetes. Iranian biomedical journal. 2018;22(5):303.

31. Asadi F, Salehnasab C, Ajori L. Supervised algorithms of machine learning for the prediction of cervical cancer. Journal of biomedical physics & engineering. 2020;10(4):513.

32. Farran B, Channanath A, Thanaraj TA. Use of non-invasive parameters and machine-learning algorithms for predicting future risk of type 2 diabetes: a retrospective cohort study of health data from Kuwait. Frontiers in endocrinology. 2019;10:436654.

33. Farran B, Channanath AM, Behbehani K, Thanaraj TA. Predictive models to assess risk of type 2 diabetes, hypertension and comorbidity: machine-learning algorithms and validation using national health data from Kuwait—a cohort study. BMJ open. 2013;3(5):e002457.

34. ForouzeshFar P, Safaei AA, Ghaderi F, Hashemikamangar SS. Dental Caries diagnosis from bitewing images using convolutional neural networks. BMC Oral Health. 2024;24(1):211.

35. Fryan LHA, Alazzam MB, editors. Survival Analysis of Oncological Patients Using Machine Learning Method. Healthcare; 2022: MDPI.

36. Ghany KKA, Zawbaa HM, Sabri HM. COVID-19 prediction using LSTM algorithm: GCC case study. Informatics in Medicine Unlocked. 2021;23:100566.

37. Gholami H, Mohammadifar A, Behrooz RD, Kaskaoutis DG, Li Y, Song Y. Intrinsic and extrinsic techniques for quantification uncertainty of an interpretable GRU deep learning model used to predict atmospheric total suspended particulates (TSP) in Zabol, Iran during the dusty period of 120-days wind. Environmental Pollution. 2024;342:123082.

38. Giannakopoulos K, Kavadella A, Aaqel Salim A, Stamatopoulos V, Kaklamanos EG. Evaluation of the performance of generative AI large language models ChatGPT, Google Bard, and Microsoft Bing Chat in supporting evidence-based dentistry: Comparative mixed methods study. Journal of medical internet research. 2023;25:e51580.

39. Göksel P, Oban V, Dikeç G, Usta MB, barış Usta M. Qualitative and artificial intelligence-based sentiment analysis of Turkish twitter messages related to autism spectrum disorders. Cureus. 2023;15(5).

40. Günal E, Budak M, Kılıç M, Cemek B, Sırrı M. Combining spatial autocorrelation with artificial intelligence models to estimate spatial distribution and risks of heavy metal pollution in agricultural soils. Environmental Monitoring and Assessment. 2023;195(2):317.

41. Habib M, Faris M, Qaddoura R, Alomari M, Alomari A, Faris H. Toward an automatic quality assessment of voice-based telemedicine consultations: A deep learning approach. Sensors. 2021;21(9):3279.

42. Habibzadeh A, Khademolhosseini S, Kouhpayeh A, Niakan A, Asadi MA, Ghasemi H, et al. Machine learning‐based models to predict the need for neurosurgical intervention after moderate traumatic brain injury. Health Science Reports. 2023;6(11):e1666.

43. Hameed Abdulkareem K, Awad Mutlag A, Musa Dinar A, Frnda J, Abed Mohammed M, Hasan Zayr F, et al. Smart healthcare system for severity prediction and critical tasks management of COVID-19 patients in IoT-fog computing environments. Computational Intelligence and Neuroscience. 2022;2022.

44. Hammoudi Halat D, Abdel-Salam A-SG, Bensaid A, Soltani A, Alsarraj L, Dalli R, Malki A. Use of machine learning to assess factors affecting progression, retention, and graduation in first-year health professions students in Qatar: a longitudinal study. BMC Medical Education. 2023;23(1):909.

45. Hassanzadeh R, Farhadian M, Rafieemehr H. Hospital mortality prediction in traumatic injuries patients: comparing different SMOTE-based machine learning algorithms. BMC medical research methodology. 2023;23(1):101.

46. Hosseini Sarkhosh SM, Esteghamati A, Hemmatabadi M, Daraei M. Predicting diabetic nephropathy in type 2 diabetic patients using machine learning algorithms. Journal of Diabetes & Metabolic Disorders. 2022;21(2):1433-41.

47. Huang W, Ao S, Han D, Liu Y, Liu S, Huang Y. Data-driven and machine-learning methods to project coronavirus disease 2019 pandemic trend in Eastern Mediterranean. Frontiers in public health. 2021;9:602353.

48. Jamei M, Ahmadianfar I, Karbasi M, Jawad AH, Farooque AA, Yaseen ZM. The assessment of emerging data-intelligence technologies for modeling Mg+ 2 and SO4− 2 surface water quality. Journal of environmental management. 2021;300:113774.

49. John M, Shaiba H. Main factors influencing recovery in MERS Co-V patients using machine learning. Journal of infection and public health. 2019;12(5):700-4.

50. Kashef A, Khatibi T, Mehrvar A. Prediction of cranial radiotherapy treatment in pediatric acute lymphoblastic leukemia patients using machine learning: A case study at MAHAK hospital. Asian Pacific Journal of Cancer Prevention: APJCP. 2020;21(11):3211.

51. Keser G, Bayrakdar İŞ, Pekiner FN, Çelik Ö, Orhan K. A deep learning algorithm for classification of oral lichen planus lesions from photographic images: A retrospective study. Journal of Stomatology, Oral and Maxillofacial Surgery. 2023;124(1):101264.

52. Khan W, Zaki N, Masud MM, Ahmad A, Ali L, Ali N, Ahmed LA. Infant birth weight estimation and low birth weight classification in United Arab Emirates using machine learning algorithms. Scientific reports. 2022;12(1):12110.

53. Khounraz F, Khodadoost M, Gholamzadeh S, Pourhamidi R, Baniasadi T, Jafarbigloo A, et al. Prognosis of COVID‐19 patients using lab tests: A data mining approach. Health Science Reports. 2023;6(1):e1049.

54. Koç E, Türkoğlu M. Forecasting of medical equipment demand and outbreak spreading based on deep long short-term memory network: the COVID-19 pandemic in Turkey. Signal, image and video processing. 2022:1-9.

55. Li J, Garshick E, Hart JE, Li L, Shi L, Al-Hemoud A, et al. Estimation of ambient PM2. 5 in Iraq and Kuwait from 2001 to 2018 using machine learning and remote sensing. Environment International. 2021;151:106445.

56. Li J, Kang C-M, Wolfson JM, Alahmad B, Al-Hemoud A, Garshick E, Koutrakis P. Estimation of fine particulate matter in an arid area from visibility based on machine learning. Journal of exposure science & environmental epidemiology. 2022;32(6):926-31.

57. Mahboobi H, Shakiba A, Mirbagheri B. Improving groundwater nitrate concentration prediction using local ensemble of machine learning models. Journal of Environmental Management. 2023;345:118782.

58. Marzouk M, Elshaboury N, Abdel-Latif A, Azab S. Deep learning model for forecasting COVID-19 outbreak in Egypt. Process Safety and Environmental Protection. 2021;153:363-75.

59. Mehrnoush V, Ranjbar A, Farashah MV, Darsareh F, Shekari M, Jahromi MS. Prediction of postpartum hemorrhage using traditional statistical analysis and a machine learning approach. AJOG Global Reports. 2023;3(2):100185.

60. Mohammadi G, Looha MA, Pourhoseingholi MA, Tavirani MR, Sohrabi S, Khaneh AZS, et al. Classification and Diagnostic Prediction of Colorectal Cancer Mortality Based on Machine Learning Algorithms: A Multicenter National Study. Asian Pacific Journal of Cancer Prevention: APJCP. 2024;25(1):333.

61. Mohammadnezhad K, Sahebi MR, Alatab S, Sadjadi A. Modeling Epidemiology Data with Machine Learning Technique to Detect Risk Factors for Gastric Cancer. Journal of Gastrointestinal Cancer. 2023:1-10.

62. Mohammadpour A, Keshtkar M, Samaei MR, Isazadeh S, Khaneghah AM. Assessing water quality index and health risk using deterministic and probabilistic approaches in Darab County, Iran; A machine learning for fluoride prediction. Chemosphere. 2024:141284.

63. Momahhed SS, Sefiddashti SE, Minaei B, Arab M. The optimal co-insurance rate for outpatient drug expenses of Iranian health insured based on the data mining method. International Journal for Equity in Health. 2024;23(1):1-12.

64. Mosayebi A, Mojaradi B, Bonyadi Naeini A, Khodadad Hosseini SH. Modeling and comparing data mining algorithms for prediction of recurrence of breast cancer. PloS one. 2020;15(10):e0237658.

65. Moslehi S, Rabiei N, Soltanian AR, Mamani M. Application of machine learning models based on decision trees in classifying the factors affecting mortality of COVID-19 patients in Hamadan, Iran. BMC Medical Informatics and Decision Making. 2022;22(1):192.

66. Mousa KM, Mousa FA, Mohamed HS, Elsawy MM. Prediction of foot ulcers using artificial intelligence for diabetic patients at Cairo university hospital, Egypt. SAGE Open Nursing. 2023;9:23779608231185873.

67. Naghavi A, Teismann T, Asgari Z, Mohebbian MR, Mansourian M, Mañanas MÁ. Accurate diagnosis of suicide ideation/behavior using robust ensemble machine learning: a university student population in the Middle East and North Africa (MENA) Region. Diagnostics. 2020;10(11):956.

68. Nopour R. Prediction of five-year survival among esophageal cancer patients using machine learning. Heliyon. 2023;9(12).

69. Nourelahi M, Dadboud F, Khalili H, Niakan A, Parsaei H. A machine learning model for predicting favorable outcome in severe traumatic brain injury patients after 6 months. Acute and critical care. 2022;37(1):45.

70. Qasrawi R, Amro M, VicunaPolo S, Al-Halawa DA, Agha H, Seir RA, et al. Machine learning techniques for predicting depression and anxiety in pregnant and postpartum women during the COVID-19 pandemic: a cross-sectional regional study. F1000Research. 2022;11.

71. Qasrawi R, Hoteit M, Tayyem R, Bookari K, Al Sabbah H, Kamel I, et al. Machine learning techniques for the identification of risk factors associated with food insecurity among adults in Arab countries during the COVID-19 pandemic. BMC public health. 2023;23(1):1805.

72. Qasrawi R, Vicuna Polo S, Abu Khader R, Abu Al-Halawa D, Hallaq S, Abu Halaweh N, Abdeen Z. Machine learning techniques for identifying mental health risk factor associated with schoolchildren cognitive ability living in politically violent environments. Frontiers in psychiatry. 2023;14:1071622.

73. Ranjbar A, Montazeri F, Farashah MV, Mehrnoush V, Darsareh F, Roozbeh N. Machine learning-based approach for predicting low birth weight. BMC Pregnancy and Childbirth. 2023;23(1):803.

74. Rashed A-HM, El-Attar NE, Abdelminaam DS, Abdelfatah M. Analysis the patients’ careflows using process mining. Plos one. 2023;18(2):e0281836.

75. Razavi-Termeh SV, Sadeghi-Niaraki A, Choi S-M. Effects of air pollution in spatio-temporal modeling of asthma-prone areas using a machine learning model. Environmental Research. 2021;200:111344.

76. Razavi-Termeh SV, Sadeghi-Niaraki A, Farhangi F, Choi S-M. Covid-19 risk mapping with considering socio-economic criteria using machine learning algorithms. International journal of environmental research and public health. 2021;18(18):9657.

77. Razavi-Termeh SV, Sadeghi-Niaraki A, Naqvi RA, Choi S-M. Dust detection and susceptibility mapping by aiding satellite imagery time series and integration of ensemble machine learning with evolutionary algorithms. Environmental Pollution. 2023;335:122241.

78. Saba AI, Elsheikh AH. Forecasting the prevalence of COVID-19 outbreak in Egypt using nonlinear autoregressive artificial neural networks. Process safety and environmental protection. 2020;141:1-8.

79. Safdari R, Maserat E, Aghdaei HA, Shalmani HM. Person centered prediction of survival in population based screening program by an intelligent clinical decision support system. Gastroenterology and Hepatology from bed to Bench. 2017;10(1):60.

80. Salehi M, Ghahari S, Hosseinzadeh M, Ghalichi L. Domestic violence risk prediction in Iran using a machine learning approach by analyzing Persian textual content in social media. Heliyon. 2023;9(5).

81. Sallam M, Al-Salahat K, Al-Ajlouni E. ChatGPT performance in diagnostic clinical microbiology laboratory-oriented case scenarios. Cureus. 2023;15(12).

82. Sancar N, Tabrizi SS. Machine learning approach for the detection of vitamin D level: a comparative study. BMC Medical Informatics and Decision Making. 2023;23(1):219.

83. Saybani MR, Shamshirband S, Hormozi SG, Wah TY, Aghabozorgi S, Pourhoseingholi MA, Olariu T. Diagnosing tuberculosis with a novel support vector machine-based artificial immune recognition system. Iranian Red Crescent medical journal. 2015;17(4).

84. Saybani MR, Shamshirband S, Golzari S, Wah TY, Saeed A, Mat Kiah ML, Balas VE. RAIRS2 a new expert system for diagnosing tuberculosis with real-world tournament selection mechanism inside artificial immune recognition system. Medical & biological engineering & computing. 2016;54:385-99.

85. Sayed GI, Solyman M, El Gedawy G, Moemen YS, Aboul-Ella H, Hassanien AE. Circulating miRNA’s biomarkers for early detection of hepatocellular carcinoma in Egyptian patients based on machine learning algorithms. Scientific Reports. 2024;14(1):4989.

86. Seyedtabib M, Kamyari N. Predicting polypharmacy in half a million adults in the Iranian population: comparison of machine learning algorithms. BMC medical informatics and decision making. 2023;23(1):84.

87. Shaibani MJ, Emamgholipour S, Moazeni SS. Investigation of robustness of hybrid artificial neural network with artificial bee colony and firefly algorithm in predicting COVID-19 new cases: case study of Iran. Stochastic Environmental Research and Risk Assessment. 2022;36(9):2461-76.

88. Sharifi-Kia A, Nahvijou A, Sheikhtaheri A. Machine learning-based mortality prediction models for smoker COVID-19 patients. BMC Medical Informatics and Decision Making. 2023;23(1):129.

89. Sheikhtaheri A, Zarkesh MR, Moradi R, Kermani F. Prediction of neonatal deaths in NICUs: development and validation of machine learning models. BMC medical informatics and decision making. 2021;21:1-14.

90. Shirmohammadi‐Khorram N, Tapak L, Hamidi O, Maryanaji Z. A comparison of three data mining time series models in prediction of monthly brucellosis surveillance data. Zoonoses and public health. 2019;66(7):759-72.

91. Shogrkhodaei SZ, Razavi-Termeh SV, Fathnia A. Spatio-temporal modeling of PM2. 5 risk mapping using three machine learning algorithms. Environmental Pollution. 2021;289:117859.

92. Shojaee-Mend H, Velayati F, Tayefi B, Babaee E. Prediction of Diabetes Using Data Mining and Machine Learning Algorithms: A Cross-Sectional Study. Healthcare Informatics Research. 2024;30(1):73.

93. Simsekler MCE, Alhashmi NH, Azar E, King N, Luqman RAMA, Al Mulla A. Exploring drivers of patient satisfaction using a random forest algorithm. BMC Medical Informatics and Decision Making. 2021;21(1):157.

94. Soltani M, Farahmand M, Pourghaderi AR. Machine learning-based demand forecasting in cancer palliative care home hospitalization. Journal of Biomedical Informatics. 2022;130:104075.

95. Talebi R, Celis-Morales CA, Akbari A, Talebi A, Borumandnia N, Pourhoseingholi MA. Machine learning-based classifiers to predict metastasis in colorectal cancer patients. Frontiers in Artificial Intelligence. 2024;7.

96. Tao H, Jawad AH, Shather A, Al-Khafaji Z, Rashid TA, Ali M, et al. Machine learning algorithms for high-resolution prediction of spatiotemporal distribution of air pollution from meteorological and soil parameters. Environment international. 2023;175:107931.

97. Torad AA, Shamy F, Kadry AM, Ahmed ZS. Using Machine Learning Models To Investigate The Relationship Between Corporeal Workload And Clinical And Epidemiological Features Of Patients Infected With COVID-19 In Egypt. JPMA The Journal of the Pakistan Medical Association. 2023;73(4):S242-S6.

98. Turk G, Ozdemir M, Zeydan R, Turk Y, Bilgin Z, Zeydan E. On the identification of thyroid nodules using semi‐supervised deep learning. International Journal for Numerical Methods in Biomedical Engineering. 2021;37(3):e3433.

99. Uyar A, Bener A, Ciray HN. Predictive modeling of implantation outcome in an in vitro fertilization setting: an application of machine learning methods. Medical Decision Making. 2015;35(6):714-25.

100. Zhang T, Li Y, Wang M. Remote sensing-based prediction of organic carbon in agricultural and natural soils influenced by salt and sand mining using machine learning. Journal of Environmental Management. 2024;352:120107.

1. Accuracy, Coverage, Objectivity, Date, Significance [↑](#footnote-ref-1)
